# The BAF A12T mutation disrupts lamin A/C interaction, impairing robust repair of nuclear envelope ruptures in Nestor–Guillermo progeria syndrome cells

**DOI:** 10.1093/nar/gkac726

**Published:** 2022-08-30

**Authors:** Anne Janssen, Agathe Marcelot, Sophia Breusegem, Pierre Legrand, Sophie Zinn-Justin, Delphine Larrieu

**Affiliations:** Department of Clinical Biochemistry, Cambridge Biomedical Campus, Cambridge Institute for Medical Research, University of Cambridge, Cambridge CB2 0XY, UK; Institute for Integrative Biology of the Cell (I2BC), CEA, CNRS, Université Paris-Sud, Université Paris-Saclay, Gif-sur-Yvette Cedex 91190, France; Department of Clinical Biochemistry, Cambridge Biomedical Campus, Cambridge Institute for Medical Research, University of Cambridge, Cambridge CB2 0XY, UK; Synchrotron SOLEIL, HelioBio group, L’Orme des Merisiers, Gif sur-Yvette 91190, France; Institute for Integrative Biology of the Cell (I2BC), CEA, CNRS, Université Paris-Sud, Université Paris-Saclay, Gif-sur-Yvette Cedex 91190, France; Department of Clinical Biochemistry, Cambridge Biomedical Campus, Cambridge Institute for Medical Research, University of Cambridge, Cambridge CB2 0XY, UK

## Abstract

Nestor–Guillermo progeria syndrome (NGPS) is caused by a homozygous alanine-to-threonine mutation at position 12 (A12T) in barrier-to-autointegration factor (BAF). It is characterized by accelerated aging with severe skeletal abnormalities. BAF is an essential protein binding to DNA and nuclear envelope (NE) proteins, involved in NE rupture repair. Here, we assessed the impact of BAF A12T on NE integrity using NGPS-derived patient fibroblasts. We observed a strong defect in lamin A/C accumulation to NE ruptures in NGPS cells, restored upon homozygous reversion of the pathogenic BAF A12T mutation with CRISPR/Cas9. By combining *in vitro* and cellular assays, we demonstrated that while the A12T mutation does not affect BAF 3D structure and phosphorylation by VRK1, it specifically decreases the interaction between BAF and lamin A/C. Finally, we revealed that the disrupted interaction does not prevent repair of NE ruptures but instead generates weak points in the NE that lead to a higher frequency of NE re-rupturing in NGPS cells. We propose that this NE fragility could directly contribute to the premature aging phenotype in patients.

## INTRODUCTION

The nuclear envelope (NE) is a critical double membrane structure that surrounds and encloses the nucleus, maintaining the organization of the chromatin (1,2), controlling nucleocytoplasmic transport and allowing the transduction of mechanical signals from the cytoplasm into the nucleus (3,4). The NE is made of the inner and outer nuclear membranes (INM and ONM). The lamina, which is a meshwork of intermediate filaments of A-type (lamins A and C, encoded by *LMNA*) and B-type (lamin B1 and B2, encoded by *LMNB1* and *LMNB2*, respectively) lamin proteins, lies at the nucleoplasmic side of the INM (5). Lamin filaments interact with LEM (LAP2-emerin-MAN1) domain proteins that are embedded in the INM, with chromatin and with other nuclear proteins. These interactions play critical roles in maintaining the structural integrity of the nucleus (6,7).

The importance of the lamina is evident by the numerous diseases arising from mutations in *LMNA* or in genes encoding for NE-associated proteins. These mutations compromise the integrity of the lamina and of the NE, causing a range of laminopathies (8,9) including premature aging syndromes, muscular dystrophies and neuropathies (10–12). One of the consequences of NE destabilization is the appearance of NE ruptures that cause loss of nuclear compartmentalization. This has been observed in laminopathy patient cells and animal models (13–15), in cells undergoing a viral infection (16,17) or lacking specific components of the lamina (13,18). Additionally, mechanical stress can cause NE rupture, for example *in vivo* when cells are migrating through dense tissues (19–21), or *in vitro* when cells are cultured in 2D on stiff substrates (22).

A NE rupture is typically preceded by the formation of a gap in the nuclear lamina, generating a weak point at the NE, more prone to deformation by mechanical stress (23,24). This allows the formation of a protrusion of the nuclear membrane that under continued mechanical stress will grow and eventually rupture (19,21,25). The exposure and leakage of the nuclear content—including the chromosomal DNA—into the cytoplasmic compartment can cause DNA damage (19,20,26) and activation of innate immune signalling pathways, such as cGAS/STING that can trigger inflammation (19,20,27,28). Importantly, the cells are able to detect and reseal a NE rupture within minutes, through the recruitment of several proteins to the site of rupture. These include barrier-to-autointegration factor (BAF), LEM domain proteins (including emerin) and ESCRT-III components (19,20,29–31). Finally, lamin A/C accumulates at the sites of ruptures, leaving behind what was defined as a lamin ‘scar’ (20,21,24), whose role remains unclear.

BAF is a small (89 amino acid) protein that localizes to the nucleus, at the NE and in the cytoplasm. BAF dimerization allows for its binding to LEM domain proteins and the Ig-fold domain of lamin A/C (32,33), while each individual subunit can bind dsDNA (34,35). BAF has been previously involved in the regulation of transcription, viral defence and postmitotic nuclear envelope reassembly (5,36–38). More recently, BAF was found to play a direct role in repairing ruptures of the NE by recruiting LEM domain proteins and membranes to the sites of rupture (29,31).

The interest around the function of BAF at the NE has grown since an alanine to threonine homozygous missense mutation at position 12 (Ala12Thr–BAF A12T) was identified as the cause of a premature aging disorder termed Nestor–Guillermo Progeria Syndrome (NGPS) (39–41). So far, only three NGPS patients have been identified and they all carry the same homozygous A12T mutation, inheriting one mutated copy from each of their parents, both carriers of a heterozygous BAF A12T mutation and devoid of disease. On the contrary to the classic Hutchinson–Gilford progeria syndrome (HGPS), caused by heterozygous *LMNA* mutations (11,42), NGPS patients live over 30 years and do not display any vascular or cardiovascular dysfunction. Instead, in addition to ageing phenotypes including alopecia, lipodystrophy and joint stiffness, they present with severe osteolysis and skeletal deformation that is the primary cause of concern in these patients.

The mechanism by which the BAF A12T mutation causes these detrimental effects remains unclear. Here, we set out to characterize the effects of this mutation on NE integrity in NGPS-derived patient cells. To this aim, we engineered the first NGPS isogenic cell lines by correcting the homozygous BAF A12T mutation in NGPS patient cells using CRISPR-Cas9 mediated genome editing. We observed that the BAF A12T mutation in NGPS patient cells prevents the recruitment of A-type lamins to NE rupture sites, a phenotype that was ameliorated upon correction of the mutation. By combining biophysical analysis and cell biology assays, we showed that while not affecting BAF structure, the A12T mutation significantly decreases the binding affinity of BAF to lamin A/C. Finally, we showed that in a disease context, BAF A12T causes reduced recruitment of A-type lamins to the sites of NE rupture. While this did not prevent or delay NE repair, this led to enhanced nuclear fragility, characterized by more frequent NE re-rupturing.

## MATERIALS AND METHODS

### Cell culture

Cells were cultured in Dulbecco’s modified Eagle’s medium containing 10% foetal calf serum and penicillin/streptomycin. Cells were maintained at 37°C and 5% CO_2_. Control human fibroblast cell line was derived from AG10803 immortalized with SV40LT and TERT, NGPS1 and NGPS2 were derived from NGPS5796 and NGPS5787, respectively, and were immortalized with SV40LT and TERT. These immortalized cell lines were a gift from Carlos López-Otín.

### Generation of stable cell lines

Stable cell lines expressing FLAG-BAFWT and FLAG-BAFA12T were generated using a PiggyBac system. First, BAF WT and BAF A12T sequence were PCR-amplified from GFP-BAF vectors (kind gift from Cristina Capanni) and inserted into a FLAG vector. Piggybac vectors containing FLAG-BAF WT and FLAG BAF A12T were subsequently assembled by PCR amplification followed by Gibson assembly using HiFi DNA assembly cloning kit (New England Biolabs, #E5520S). FLAG-BAF was cloned into a BamHI and AfeI digested piggybacV1_CMV, a custom piggyback vector containing a CMV promotor and hygromycin resistance gene (kind gift from Jonathon Nixon-Abell). Stable cell lines expressing GFP-BAF WT and GFP-BAF A12T were generated using a PiggyBac system. EGFP-linker-BAF was first assembled in another vector and contains a short (GGGGS)_2_ linker. Piggybac vectors containing GFP-BAF WT and GFP-BAF A12T were subsequently assembled by PCR amplification followed by Gibson assembly into a BamHI and AfeI digested piggybacV1_CMV. Piggybac vector containing GFP-NLS was assembled by PCR amplification of 3xGFP-NLS followed by Gibson assembly using HiFi DNA assembly cloning kit (New England Biolabs, #E5520S). The 3xGFP-NLS was described previously (43) and contains cycle3GFP fused to EGFP, an NLS, and a second EGFP in a pcDNA backbone (kind gift from Emily Hatch). 3xGFP NLS was cloned into a BamHI and AfeI digested piggybacV1_CMV.

Stable cell lines were generated by transfecting cells with the piggybacV1 plasmid together with a second plasmid containing the PiggyBac transposase (Jonathon Nixon-Abell) under an EF1alpha promoter using lipofectamine 3000 or Transit2020, according to manufacturer’s protocol. Cells were grown on hygromycin selection (100 μg/ml) the next day and were maintained in hygromycin containing medium.

### Reversion of the BAF A12T mutation using CRISPR-Cas9 gene editing

To reverse the A12T mutation in *BANF1*, we used NGPS2 cells as they grew clones more easily from single cells. We used a previously described strategy that combines an All-in-One Cas9^D10A^ nickase vector with enrichment by fluorescence-activated cell sorting to enrich for transfected cells (44). A pair of guide RNAs (sgRNAs) was designed to target the DNA on opposing strands surrounding the *BANF1* mutation in intron 1. The sgRNAs were then cloned into the All-in-One Cas9^D10A^ nickase vector using DNA oligos (Sigma-Aldrich) using the BsaI and BbsI recognition sites. Several sgRNA pairs were originally designed using the CRISPR design tool in Benchling. After optimization, the sgRNA sequences: GCCCATGGGGGAGAAGCCAG and GAAGTCTCGGTGCTTTTGGG were used for CRISPR targeting in combination with a PAGE-purified ssODN of 200 bp long (Integrated DNA Technologies). The ssODN contains the BANF1 WT genomic sequence to reverse the A12T mutation and in addition has silent mutations to mutate the PAM sites to prevent Cas9 recutting and a mutation in an NcoI restriction site to facilitate clone screening by digestion of PCR products. Full ssODN sequence:AGAAGTTCCAGGTCTTCAGCCCTAATCTGCCTTTTTTTTGGGATTCCTAGATTAAGCCTGATCAAGATGACAACATCCCAAAAGCACCGAGACTTCGTGGCAGAGCCTATGGGGGAGAAGCCAGTCGGGAGCCTGGCTGGGATTGGTGAAGTCCTGGGCAAGAAGCTGGAGGAAAGGGGTTTTGACAAGGTGTGGGGTGG.

Cells were transfected with the All-in-One vector and the ssODN using Transit2020. GFP positive cells were sorted the next day either directly into 96-well plates or as a polyclonal population for manual seeding into 96-well plates. Genomic DNA of expanded clones was isolated using QuickExtract DNA extraction solution (Lucigen, QE0905T). PCR was performed by amplifying a 241 bp area surrounding the mutation site, followed by restriction digestion using NcoI to screen for positive clones.

### RNA isolation and cDNA sequencing

RNA isolation was performed starting with a confluent 10 cm dish and using the Monarch total RNA miniprep kit for RNA isolation (NEB, #T2010S). RNA was stored at −80°C. RT-PCR was performed using a One-Step RT-PCR kit (Qiagen, #210210) using the following primers: Forward: AAAGCACCGAGACTTCGTGG and Reverse: AAGGCATCCGAAGCAGTCC. The reaction was set up according to manufacturer’s protocol. The generated cDNA products were gel purified using Qiaquick Gel Extraction kit (Qiagen, #28704) and sent for sequencing.

### Immunoblotting

Cells were washed with ice-cold PBS, lysed in Laemmli buffer (4% SDS, 20% glycerol, and 120 mM Tris-HCl [pH 6.8]) and then incubated for 5 min at 95°C. The DNA was sheared by syringing the lysates 10 times through a 25-gauge needle. Absorbance at 280 nm was measured (NanoDrop; Thermo Fisher Scientific) to determine protein concentration. Samples were prepared using Protein Sample Loading Buffer (LI-COR, #928-40004) and DTT (final concentration 50 mM) and heated at 95°C for 5 min. Proteins were separated using NuPAGE 4-12% Bis-Tris gels (ThermoFisher) and NuPAGE MES SDS running buffer (Thermo Fisher, #NP0002) and transferred to nitrocellulose membranes for immunoblotting. Membranes were blocked in 5% milk PBS and incubated overnight at 4°C with primary antibodies. Next day, membranes were incubated for 1 h at room temperature with IRDye-conjugated secondary antibodies (LI-COR) and scanned on an Odyssey imaging system. The following primary antibodies were used: mouse anti-Tubulin (Sigma Aldrich, #T9026, 1/2000), mouse anti-lamin A/C (Santa Cruz, #sc-7292, 1/500), mouse anti-lamin B1 (Santa Cruz, #sc-365214, 1/1000), rabbit anti-emerin (Proteintech, #10351-1-AP, 1/1000), rabbit anti-H3K9me3 (Abcam, #ab8898, 1/1000), rabbit anti-BAF (ProSci, #4019, 1/500), mouse anti-γH2AX (Millipore, #05-636-I, 1/500) and rabbit anti-H2AX (Bethyl, A300-083A-T, 1/1000).

### Immunofluorescence

Cells were fixed at room temperature for 10 min with 4% PFA. Cells were washed in PBS, permeabilized using 0.2% Triton-X100, and blocked using 3% bovine serum albumin (BSA) in PBS for 30 min. Cells were incubated overnight at 4°C or for 1 h at RT in 3% BSA PBS containing primary antibody. Cells were washed using PBS and incubated for 1 h at room temperature with secondary antibody in 3% BSA PBS. Cells were washed in PBS and mounted using Prolong Gold (Thermo Fischer). The following primary antibodies were used: rabbit anti-BAF (Abcam, ab129184, 1/200), mouse anti-lamin A/C (Santa Cruz, sc-376248, 1/500), mouse anti-lamin A/C (Santa Cruz, sc-7292, 1/500), rabbit anti-emerin (Proteintech, 10351-1-AP, 1/500), mouse anti-lamin B1 (Santa Cruz, sc-365214, 1/500), mouse anti-yH2AX (Millipore, 05-636-I, 1/200), rabbit anti-sun1 (Abcam, ab124770, 1/100) and mouse anti-FLAG (Sigma Aldrich, F1804, 1/1000). Images were taken on Zeiss Axio Imager Z2 using a 63× oil immersion objective (Plan APO, NA 1.4, Zeiss).

### Image processing and analysis

Quantitative analysis of nuclear/cytoplasmic ratio’s, nuclear size and recruitment of proteins to blebs was performed using CellProfiler V4.1.3. Custom pipelines will be made available on request.

Nuclear/cytoplasmic ratio: Nuclear object was identified based on DAPI channel. The cytoplasm object was subsequently generated by expansion of the nuclear object by 50 pixels and subtraction of the nucleus to create a cytoplasmic ring. The intensity of additional channels in the nuclear and cytoplasmic object were measured. In addition, the area of the nuclear object was measured. Data were transferred to Microsoft Excel for calculation of the nuclear/cytoplasmic ratio and subsequently plotted and analysed using GraphPad Prism 9.

Bleb analysis (45): Chromatin object was identified based on DAPI staining. A lamin B1 object was identified and subtraction of both areas identifies the bleb object as it is devoid of lamin B1. Subsequently, bleb areas are filtered to exclude small identified regions or pixels at the edge of the nuclei. Size, shape and number of bleb areas are measured as well as intensity of any additional channels at the bleb regions.

### Quantitation of γH2AX foci by automated microscopy

Cells (Control, NGPS1, NGPS2, NGPS2 WT clone1 or clone2) were plated in 96-well Viewplates (Perkin Elmer, Beaconsfield, UK) and allowed to grow to 80% confluency over 48 h in a 37°C incubator. All subsequent steps were carried out at room temperature. Cells were washed once with PBS before fixing with 4% paraformaldehyde (AR1068, Boster) for 15 min, followed by permeabilisation with 0.2% Triton X-100 (AppliChem) in PBS for 12 min. Unspecific antibody binding was blocked by incubation in 5% bovine serum albumin (Sigma), 0.2% Tween-20 (Fisher) in PBS for 30 min. Primary and secondary antibodies were diluted in this blocking buffer. Antibody incubations were for 1.5 h. Mouse anti-phospho(S139)-histone H2AX (Millipore, #05-636-I, 1/300) primary antibody was used. DAPI (EMP Biotech, 0.2 μg/ml) was added together with the secondary antibody Alexa Fluor 568 goat anti-mouse IgG_1_ (LifeTechnologies, #A21124, 1/500). Cells were washed with PBS once after the primary antibody incubation and twice after the secondary antibody incubation, and stored in PBS at 4°C until imaged. Images were acquired on a CellInsight CX7 high-content microscope (Thermo Fisher) using a 20× objective (NA 0.45, Olympus). DAPI was used for automated focussing and object detection, and 1000 objects (nuclei) were imaged in three wells for each cell line. DAPI intensity thresholds were used to exclude mitotic cells from the analysis. Phospho(S139)-histone H2AX foci were detected using the spot detection algorithm in the HCS Studio software. Spots were detected after background correction using the box method and a fixed intensity threshold. Data were exported to Excel and average number of spots per nuclei were calculated and plotted in Graphpad Prism. Results from the technical repeats were averaged, and three biological repeats were carried out.

### Proximity ligation assays (PLA)

Proximity ligation assays (PLA) were used to detect interaction between endogenous lamin A/C and BAF in control, NGPS2 and NGPS2 clone1 cells. Cells were seeded on 12 mm coverslips and fixed for 10 min with 4% PFA at room temperature and permeabilized using 0.2% Triton-X100. Cells were blocked for 1.5 h at room temperature using manufacturers blocking solution and primary antibody incubation, DuoLink Probe incubation, ligation and amplification steps were all carried out according to manufacturer’s protocol (DuoLink PLA assay kit, #DUO92008, Sigma Aldrich). Primary antibodies used were rabbit anti-BAF (Abcam, ab129184, 1/200) and mouse anti-lamin A/C (Santa Cruz, sc-376248, 1/1000). Duolink In Situ PLA probe anti-rabbit PLUS, Duolink In Situ PLA probe anti-mouse MINUS and Duolink Amplification Red were used. Cells were mounted in Duolink Mounting Media with DAPI. Confocal microscopy image acquisition was performed using LSM880 Laser scanning microscope (Zeiss) using a 63× oil immersion objective (Plan Apo, NA 1.4, Zeiss). Single plane images were taken except for analysis of PLA foci location, Z-stacks were recorded, and the middle slice was eventually used for analysis.

Quantitative analysis of PLA foci was performed using CellProfiler V4.1.3. In brief, first the nuclear object was detected based on DAPI signal after which PLA foci were detected in the nuclear object using thresholding. The number of PLA foci per nucleus was plotted for three independent experiments and the mean was indicated. Statistical analysis was performed using GraphPad PRISM 9. For analysis of the location of PLA foci, the middle slice from the recorded Z-stack was used. In brief, the nucleus was detected based on DAPI signal after which two outer rings were identified by shrinking the nucleus by 10 pixels. The defined outer nuclear circles and inner objects were used to identify the number of PLA foci in these areas. These numbers were then normalized for the surface area, and the fraction of foci in the outer rings and the centre was calculated.

### Live cell imaging and analysis

Live-cell imaging of spontaneous NE ruptures was performed with an AxioObserver Z1 Inverted microscope (Zeiss) equipped with a HXP 120V high-pressure metal-halide lightsource. For imaging of spontaneous ruptures, cells were imaged in 4-well μ-slides (Ibidi, #80426) in FluoroBrite DMEM + FBS + PSQ and were maintained at 37°C and 5% CO_2_. Cells stably expressing GFP-NLS were imaged every 3 min for 8 h using a 20× air objective (APOCHROMAT, NA 0.8, Zeiss) and an ORCA-Flash4 v2 sCMOS camera (Hamamatsu). 1/2000 SPY650 DNA (COMPANY) was added 1 h before start imaging to allow for detection of nuclei. Image analysis was performed in FIJI. For analysis of nuclear GFP-NLS intensity image sequences were cropped to individual nuclei based on the presence of rupture events. Cells that were not visible for the entire timelapse, underwent mitosis or showed severe nuclear shape abnormalities were excluded from the analysis. Cells were then automatically tracked using a dedicated script (trackRuptures_V5, (30)). Nuclei were automatically tracked based on SPY650-DNA signal. The nuclear NLS-GFP mean intensities were transferred to Microsoft Excel for further analysis. First, background subtraction was performed based on a background Region Of Interest (ROI), followed by normalization based on 5 frames before rupture occurs if available. First frame of rupture was aligned at *t* = 0. Ruptures were identified based on decreased NLS-GFP intensity and manually verified in time lapses. Ruptures were classified as major rupture events when the mean intensity loss in the nucleus was >30%. Time between ruptures after a major rupture was identified based on decrease in NLS-GFP intensity (including minor ruptures) and manually verified in time lapses. Ruptures were only included if they could be followed for at least 30 frames after the major rupture event so that they could be pooled in the overflow bin. For assessing differences in the probability of re-rupturing between Control and NGPS cell line, a Fisher’s exact test was performed. The time interval between rupture events from all replicates were pooled and we compared the number of ruptures in the >90 min category versus all other ruptures. Pooling was required due to low numbers of values across categories.

For imaging of GFP-BAF recruitment to sites of ruptures. GFP-BAF expressing cells were seeded in 35 mm Fluorodishes (#FD35, World Precision Instruments). One hour before the start of experiments, medium was replaced with CO_2_ independent medium supplemented with 1/2000 SPY650-DNA (#SC501, Spirochrome) and cells were maintained at 37°C. Compression was performed using a Dynamic Cell Confiner (4D Cell). The confinement slides (0.2 μm pillar height) and suction cup were incubated in medium 1 h before start of the experiment. Live cell imaging of GFP-BAF expressing cells was performed on the same AxioObserver Z1 Inverted microscope using a 40× objective (APOCHROMAT, NA 0.95, Zeiss). Suction cup with confinement slide was placed into the Fluorodish, and pressure was lowered to −30 mbar, which ensures the device sticks to the dish without compression. At this point, 30 s timelapse imaging of GFP and SPY650-DNA was started and at least three frames were recorded before compression. Pressure was decreased slowly to −100 mbar allowing compression of cells to 0.2 μm and was held for 1 min after which compression was slowly released by bringing the pressure back up to −30 mbar. After compression release, cells were imaged for 1 h.

### Protein constructs and expression vectors

For protein purification, we used our BAF WT construct that codes for an N-terminal tag containing 8 histidines, a TEV cleavage site, and the human BAF sequence from which all cysteines were mutated into alanines to allow for protein resistance to oxidation and thus, aggregation (32). After cleavage of the tag, the purified protein corresponds to human BAF containing the following mutations: M1G, C67A, C77A, C80A and C85A. The gene coding for BAF WT was synthesized by Genscript after codon optimization for expression in *Escherichia coli* and cloned in a pETM13 vector providing kanamycin resistance to bacteria. The vector used for BAF A12T expression was obtained by mutagenesis of the BAF WT expression vector using the Quikchange Site-Directed Mutagenesis kit (Agilent). The lamin A/C Ig-fold construct codes for a GST tag, a thrombin cleavage site, and the human lamin A/C fragment from aa 411 to aa 566. It was cloned in a pGEX vector providing an ampicillin resistance. The Vaccinia Related Kinase 1 (VRK1) expression vector is a gift from John Chodera, Nicholas Levinson and Markus Seeliger (Addgene plasmid #79684 (46)). All expression vectors were purified using the New England BioLabs kit (reference #T1010L) from 5 ml of bacteria culture.

### Protein expression and purification

All vectors were transformed in *E. coli* BL21* (DE3). The transformations were carried out by adding 100 ng of plasmid, on to about one billion of bacteria (30 μl). Vectors entry in the cells was triggered by a heat shock at 42°C during 45 s. Finally, the cells were spread on LB agar medium supplemented with the appropriated antibiotic.

Bacteria grew either in LB (Lysogeny Broth) or M9 (Minimum 9) medium depending on the needs. The M9 media were supplemented with either ^15^NH_4_Cl or natural abundance glucose. Precultures of bacteria containing the vector coding for the protein of interest were prepared in LB with antibiotic and incubated overnight at 37°C under agitation (180 rpm). Then, 20 ml of preculture was used to inoculate 800 ml of culture (LB or M9). When the OD_600nm_ reached 0.8 ± 0.1, protein expression was triggered using IPTG (isopropyl-β-D-thiogalactoside). All proteins were expressed overnight at 20°C. Cells were finally harvested by centrifugation, flashed frozen in 30 ml of lysis buffer (50 mM Tris HCl pH 8, 300 mM NaCl, 5% glycerol, 0.1% Triton X-100, 1 mM PMSF) and stored at −20°C during maximum 1–2 months.

Both BAF constructs are insoluble after overexpression in *E. coli*, so purification was performed in urea and followed by a refolding step. After sonication in lysis buffer (50 mM Tris, pH 8, 300 mM NaCl, 5% glycerol, 0.1% Triton 100X), and centrifugation at 50 000 *g* for 15 min at 4°C, the pellet was resuspended in urea purification buffer (50 mM Tris, pH 8.0, 150 mM NaCl, 8 M urea) for 20 min. Then, the sample was centrifuged again and the soluble fraction was incubated on Ni-NTA beads pre-equilibrated with urea purification buffer, for 30 min at room temperature. Ni-NTA beads were washed with the purification buffer, and the protein was eluted in 50 ml of the same buffer supplemented with 1 M imidazole. Proteins were then refolded by dialysis in BAF buffer (50 mM Tris, pH 8, 150 mM NaCl). After concentration, the histidine-tag was cleaved by the TEV protease (from a Batch of TEV purified in the lab) overnight at 4°C. The protein was separated from the TEV protease (containing a histidine tag) and its Tag by Ni-NTA affinity chromatography. Finally, a gel filtration was performed using a Superdex 200 pg HiLoad 16/600 column (GE healthcare). The final yield was typically about 0.6 mg (LB) or 0.1 mg (M9) of purified protein per liter of bacterial culture for BAF WT and twice more for BAF A12T.

For the lamin A/C Ig-fold domain, after sonication at 10°C, the supernatant was incubated 20 min at room temperature with benzonase and centrifuged at 50 000 *g* for 15 min at 4°C. The soluble extract was then supplemented with 5 mM DTT and loaded on to glutathione beads. After 1 h of incubation at 4°C, glutathione beads were washed first with 1 M NaCl buffer and then with the purification buffer (50 mM Tris, pH 7.5, 150 mM NaCl, 5 mM DTT). The GST tag was cleaved with thrombin (commercial thrombin, Sigma Aldrich), at 200 units per ml for 2 h at room temperature, then the protein was recovered in the flow-through and separated from thrombin and last contaminants using gel filtration (Superdex 200 pg HiLoad 16/600 column, GE healthcare). The final yield was typically 20 mg (LB) or 6 mg (M9) of purified protein per liter of bacterial culture.

In the case of VRK1, after sonication, the soluble extract was incubated with benzonase for 20 min at 20°C (room temperature). The lysate was then centrifuged at 50 000 *g* for 15 min at 4°C and loaded on to a 5 ml Ni-NTA column (FF crude, GE-Healthcare). The column was washed with washing buffer (50 mM Tris, pH 8.0, 1 M NaCl), re-equilibrated with purification buffer (50 mM Tris, pH 8.0, 150 mM NaCl) and eluted with an imidazole gradient (0–500 mM). After concentration to 5 ml, the histidine tag was cleaved by the TEV protease (from a batch of TEV purified in the lab) during 1 h 30 min at room temperature. Proteins were separated from the TEV protease (containing a histidine tag) by affinity chromatography, using Ni-NTA beads. Finally, last contaminants were removed by gel filtration (Superdex-200 HiLoad 16/600 column). The final yield was typically 28 mg (LB) of purified protein per liter of bacterial culture.

### X-ray crystallography

The BAF A12T-lamin A/C Ig-fold complex was recovered after ITC experiments (in 50 mM Hepes, pH 7.4, 150 mM NaCl) and concentrated to about 20 mg/ml. Crystallization experiments were carried out at the HTX Lab (EMBL Grenoble) (47). Crystals were obtained by sitting drop vapor diffusion at room temperature against reservoir containing 0.1 M bicine (pH 9) and 3 M ammonium sulfate. They were flash-frozen in liquid nitrogen and prepared for X-ray diffraction experiments using the CrystalDirect technology (48). Diffraction data were collected on the MASSIF-1 beamline (ESRF synchrotron, Grenoble, France). The 3D structure was solved by molecular replacement with Molrep software in CCP4 using the 6GHD.pdb coordinates file as starting model (49,50). The resulting model was iteratively improved by alternating manual reconstruction with the COOT software (51) and refinement with the BUSTER (52) and PHENIX REFINE softwares (53) (Supplementary Table S1). Structure coordinates were deposited to the PDB, with entry 7Z21. All structure representations and Cα RMSD calculations were performed with PyMOL (Schrodinger, LLC).

### Liquid-state nuclear magnetic resonance spectroscopy

NMR experiments were performed on 600 and 700 MHz spectrometers equipped with triple resonance cryogenic probes. The data were processed using Topspin v. 4.0.2 to v. 4.0.8 (Bruker) and analysed using Topspin 4.1.3 (Bruker) and CCPNMR 2.4 (54). Sodium trimethylsilylpropanesulfonate (DSS) was used as a chemical shift reference.

For monitoring phosphorylation of BAF WT and BAF A12T by NMR, 2D ^1^H-^15^N HSQC spectra were recorded at 303 K on a 700 MHz spectrometer. The 3 mm-diameter NMR sample tube contained 150 μM of BAF (either WT or A12T) in kinetics 40 mM HEPES, pH 7.2, 150 mM NaCl, 5 mM ATP, 5 mM MgSO_4_, 1 mM TCEP, 1× antiproteases (Roche), 95:5 H_2_O:D_2_O, and 150 nM of VRK1 kinase (molar ratio relatively to BAF: 0.1%). 2D ^1^H-^15^N NMR spectra were acquired every 15–25 min, and 1D ^1^H spectra were recorded in between to report for potential pH drifts.

To detect an interaction between the lamin A/C Ig-fold domain and BAF (either WT or A12T), 2D ^1^H-^15^N HSQC spectra were recorded on a sample containing 80 μM of ^15^N labelled lamin A/C Ig-fold and non-labeled BAF at different ratios. For the interaction with BAF WT, two spectra were acquired by adding 80 or 160 μM of BAF WT corresponding to molar ratios of 1:1 and 1:2, respectively. For the interaction with BAF A12T, two spectra were acquired: the first with 80 μM lamin A/C Ig-fold domain and 160 μM of BAF A12T, corresponding to a ratio of 1:2; the second with the lamin A/C Ig-fold domain at 160 μM and BAF WT at 160 μM, corresponding to a ratio of 1:1. The number of scans during the NMR acquisition was adjusted to obtain similar signal-to-noise ratios. All these experiments were performed in 50 mM HEPES, pH 7.4, 150 mM NaCl, 95:5 H_2_O:D_2_O, DSS, in 3 mm diameter tubes, at 293 K. Control experiments were carried out with the labelled protein at 80 μM, in the same experimental conditions.

To calculate the NMR intensity ratios *I*/*I*_o_, we started by assigning the ^1^H-^15^N HSQC signals to their corresponding lamin residues. Using a previous assignment of the Ig-fold domain (BMRB: 5224), we managed to assign the ^1^H-^15^N HSQC signals of approximately 80% of the lamin residues. The volume of each peak was measured using CCPNMR 2.4 and the reference lamin A/C Ig-fold ^1^H-^15^N HSQC spectrum at 80 μM used to obtain *I*_o_ values. When plotting intensity ratios as a function of the residue number, values corresponding to residues that could not be assigned were calculated as the average between the ratios of the previous and next residues.

### Isothermal titration calorimetry (ITC) binding assays

The interaction between lamin A/C Ig-fold domain and BAF (either WT or A12T) was assessed by ITC using a VP-ITC calorimetry system (MicroCal-Malvern Panalytical, Malvern, UK). Calorimetric titrations were performed with either 100 or 200 μM lamin A/C Ig-fold domain in the injecting syringe and either 20 μM BAF WT or 40 μM BAF A12T in the calorimetric cell, all in 50 mM Hepes, pH 7.4 and 150 mM NaCl. All measurements were performed at 288 K in order to increase the signal-to-noise ratio. For each titration, a sequence of 29 times 10 μl injections was programmed, with reference power of 10 mcal/s and spacing between injections of 180 s. Each experiment was performed twice. Data were analysed using Origin (OriginLab, Northampton, MA, USA) by setting the stoichiometry to 0.5 (assuming that a dimer of BAF binds to a monomer of lamin A/C Ig-fold domain, as observed by X-ray crystallography).

### Statistics

Statistical analysis was done using GraphPad Prism v9. Individual data points are plotted from three independent experiments. Mean or median is indicated by the black bar as indicated in the figure legends. Post-testing was performed as indicated to correct for multiple comparison. Details of statistical tests are included in the figure legends. Sample size was not predetermined and experiments were not randomized.

## RESULTS

### The phenotypes of NGPS cells are distinct from the ones of HGPS cells

**Figure 1.**
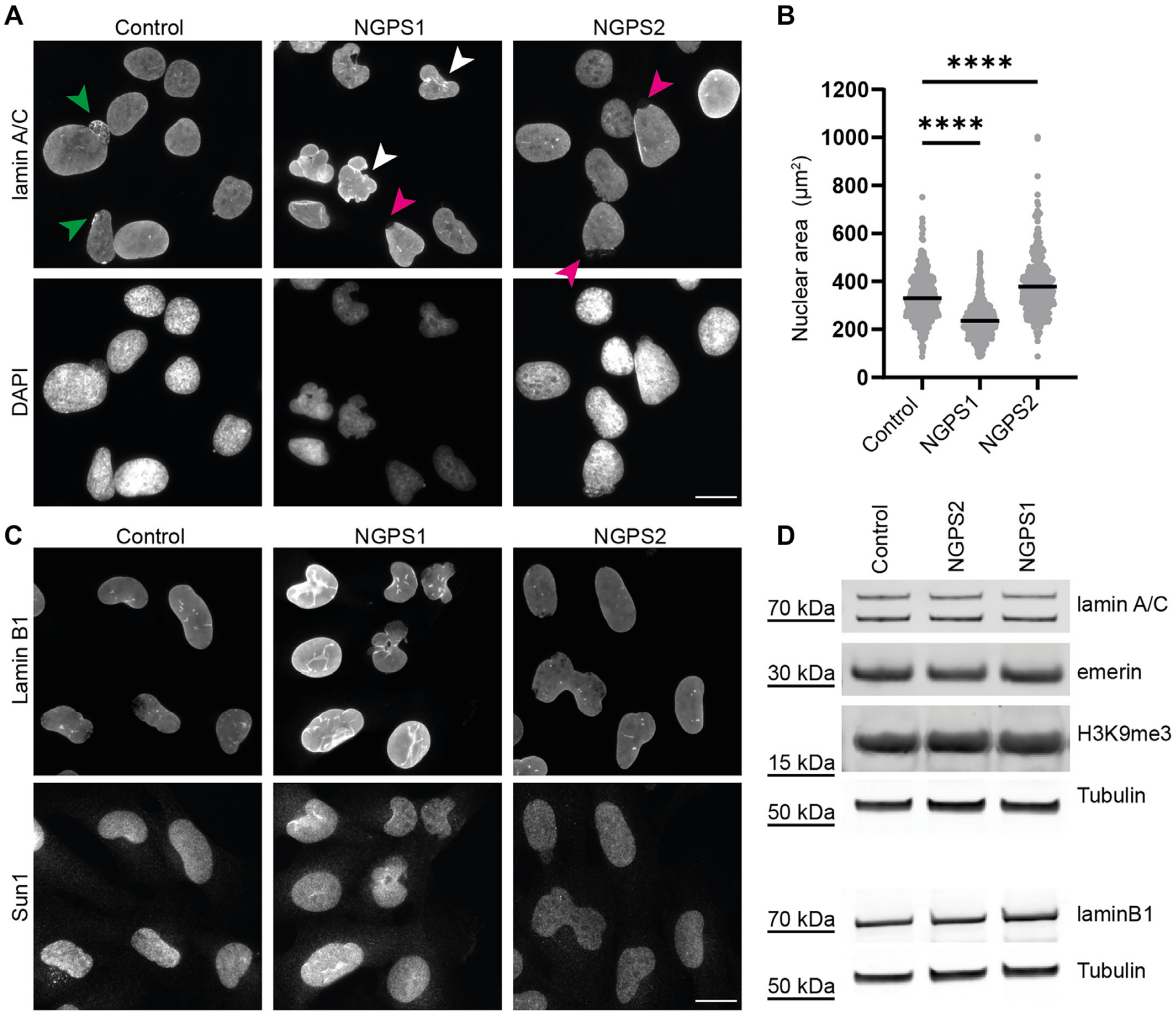
Characterization and comparison of fibroblast cell lines derived from two Nestor–Guillermo progeria patients. (**A**) Representative lamin A/C immunofluorescence images of immortalized fibroblasts obtained from an unrelated healthy donor (Control) or from two NGPS patients (NGPS1 and NGPS2). The arrows indicate areas of increased lamin intensity (green), nuclear envelope folding (white) and lamina gaps (magenta); scale bar: 20 μm. (**B**) Quantification of nuclear area based on DAPI images as presented in (A). Mean from *n* = 469, 563 and 413 nuclei for Control, NGPS1 and NGPS2, respectively. The data were collected in three independent experiments and analysed using a one-way ANOVA analysis with Šídák's multiple comparisons test (****P<0.0001). (**C**) Representative immunofluorescence images of immortalized NGPS patient and control cell fibroblasts stained for lamin B1, and Sun1; scale bar: 20 μm. (**D**) Immunoblot analysis of the indicated proteins from whole-cell lysate in Control and NGPS cells. Tubulin was used as a loading control.

To characterize NGPS patient cell phenotypes, we used hTERT immortalized skin fibroblasts of a healthy age-matched donor (control) and two NGPS patients (NGPS1 and NGPS2) (gift from C. Lopez-Otin). Microscope visualization of the nuclei in both NGPS patient cells showed clear differences between the cell lines. Cells from NGPS patients were previously reported as showing nuclear abnormalities including nuclear blebs and delocalization of emerin from the NE to the cytoplasm (40,55). Here, we observed that NGPS1 cells displayed smaller nuclei with nuclear membrane folding (white arrowheads), while NGPS2 cells had bigger nuclei, and discontinuity of the lamin A/C network (magenta arrowheads) without the presence of membrane folds (Figure 1A, B). The control cells looked more homogeneous even though they also showed some nuclear shape abnormalities with areas of lamin A/C accumulation at the NE (green arrowheads).

We further assessed several NE constituents known to be altered in HGPS, in senescent cells or in cells from normal aged individuals. We first assessed the lamin B1 expression level, as its downregulation has previously been associated with senescence (56) and observed in HGPS cells (57,58). However, NGPS cells did not show any reduction in lamin B1 (Figure 1C, D). Similarly, we did not observe any change in the expression levels of lamin A/C or emerin (Figure 1D) in these cells. In HGPS cells, the LINC (Linker of Nucleoskeleton and Cytoskeleton) complex protein SUN1 has been shown to accumulate at the NE and in the Golgi, contributing to HGPS pathogenicity (59). On the contrary, the expression and localization of SUN1 was not affected in NGPS cells (Figure 1C). Apart from NE changes, DNA damage accumulation is another marker frequently observed in various age-related diseases including HGPS (60). Perhaps surprisingly, we did not find increased DNA damage in NGPS cells—as assessed by immunofluorescence staining and immunoblotting of γH2AX, a marker of DNA double-strand breaks (Supplementary Figure S1). Finally, we investigated other potential ageing-associated changes in chromatin organization by probing for H3K9me3, a marker of transcriptionally silent heterochromatin, loss of which is another hallmark of HGPS and aging (57,61). However, H3K9me3 level was similar in control and NGPS patient cells (Figure 1D).

Together, these observations reinforce the fact that, despite the existence of premature aging features in NGPS patients, the effects of the BAF A12T mutation at the cellular level are very different from the ones caused by *LMNA* mutations in HGPS. One potential caveat here is the use of immortalized cells, which could impact on the expression level of some of these markers, however primary NGPS cells could not be obtained due to their inability to grow in culture.

### The BAF A12T mutation modifies BAF and emerin localization in NGPS cells

**Figure 2.**
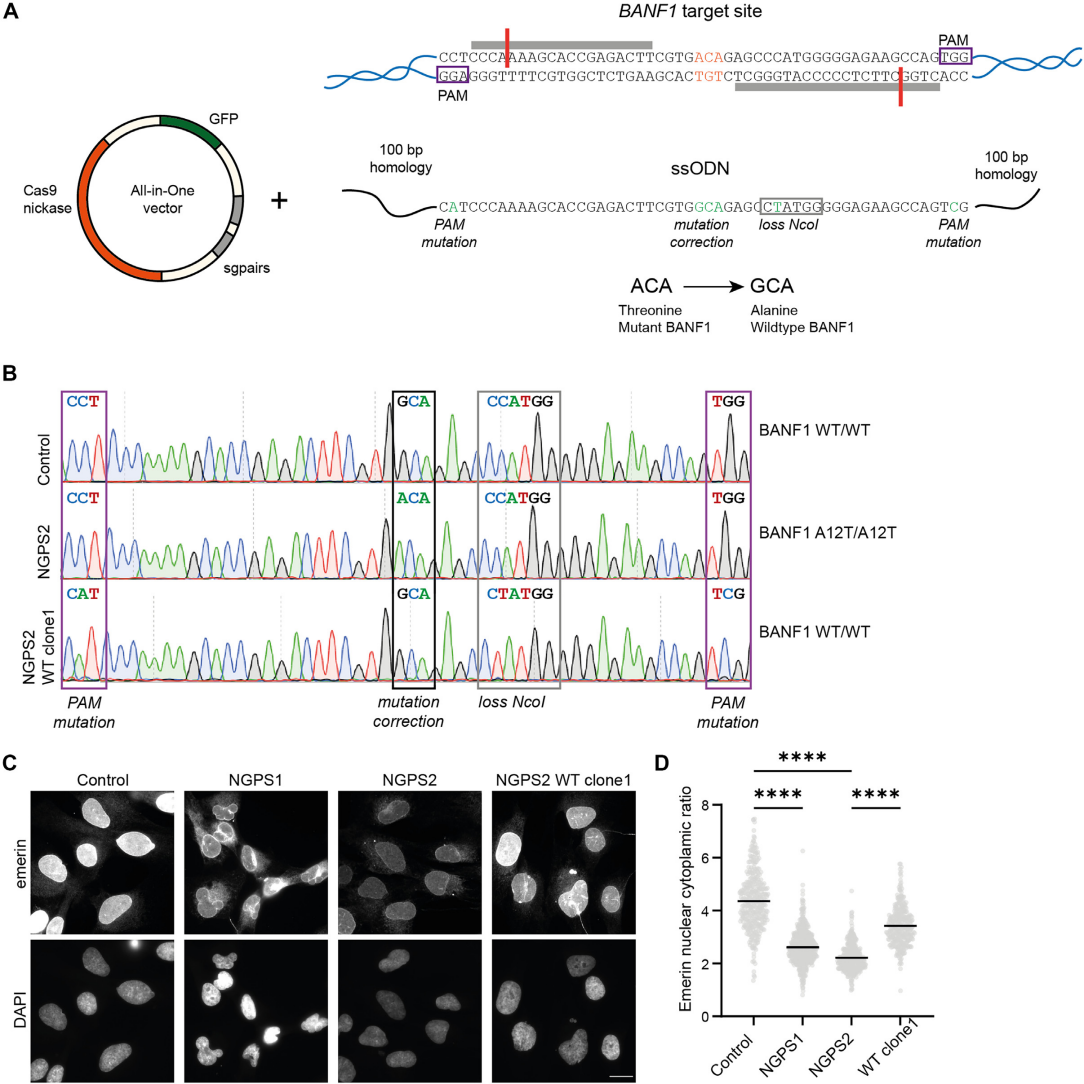
Reversion of the homozygous BAFA12T mutation in NGPS patient cells using CRISPR/Cas9. (**A**) Graphical representation of the CRISPR-Cas9 strategy showing: (top) *BANF1* sequence around the A12T mutated codon (orange), the PAM sites for Cas9 recognition (purple boxes) and the annealing sites of the single guide RNAs (sgRNAs: grey bars). Left: the all-in-one vector carrying the Cas9 nickase (orange), the sgRNA pairs targeting the *BANF1* mutation site (grey) and the Green Fluorescent Protein (GFP: used for cell sorting) was used in combination with an ssODN (bottom) carrying the correct wild-type *BANF1* sequence as a template for mutation correction through homologous recombination. (**B**) DNA sequencing traces of the *BANF1* gene around the mutation site showing the wild-type GCA codon (Alanine) in control cells, the mutated ACA (Threonine) codon in NGPS2 cells and the corrected mutation (GCA, Alanine) in one of the successfully NGPS2 derived wild-type clones (NGPS2 WT clone1). Boxes indicate the A12T mutation site (black) and additional silent mutations introduced in the PAM motifs (purple) and in the restriction enzyme NcoI site for screening (grey). (**C**) Representative immunofluorescence images of emerin in Control, NGPS patient cells and NGPS2 WT clone1; scale bar: 20 μm. (**D**) Quantification of the mean emerin nuclear to cytoplasmic ratio measured in 345, 465, 334 and 301 cells for Control, NGPS1, NGPS2 and NGPS2 WTclone1 respectively. Data are presented from three independent experiments, and the *P* value (****P<0.0001) was calculated using a one-way ANOVA analysis with Šídák’s multiple comparisons test.

Through these initial studies, we thus did not find highly consistent phenotypes associated with aging in the available patient cell lines derived from the two originally identified NGPS patients. Therefore, we decided to engineer an isogenic cell line using CRISPR-Cas9 gene editing to reverse the BAF A12T homozygous mutation in NGPS cells using an all-in-one plasmid strategy containing a Cas9 nickase, two sgRNA targeting BAF around the mutation site, and GFP for cell sorting (Figure 2A) (44). We identified two separate clones with a homozygous reversion of the A12T mutation (NGPS2 WT clone1-2) (Figure 2B and Supplementary Figure S2a). Using these isogenic cell lines, we observed that reversing the BAF A12T mutation significantly improves the emerin nuclear/cytoplasmic ratio being down to 50% of the control cells ratio in the NGPS2 cells and restored to approximately 80% of the control cells’ in the WT clone (Figure 2C, D; Supplementary Figure S2b, c), confirming that this phenotype is a direct consequence of the BAF mutation. One possible explanation for the partial rescue observed between the WT clone and the control cells could be due to differences in the basal ratio observed in cells from different individuals, as the control cells are derived from a healthy, unrelated donor.

Interestingly, *BANF1* sequencing revealed the presence of an additional and similar deletion in intron 2 in both NGPS patient cell lines (Supplementary Figure S3a). To check whether this deletion might affect RNA splicing and therefore result in an altered protein, we isolated RNA from both patient cell lines. Sequencing of the generated cDNA showed that this deletion did not affect the mRNA sequence (Supplementary Figure S3b).

**Figure 3.**
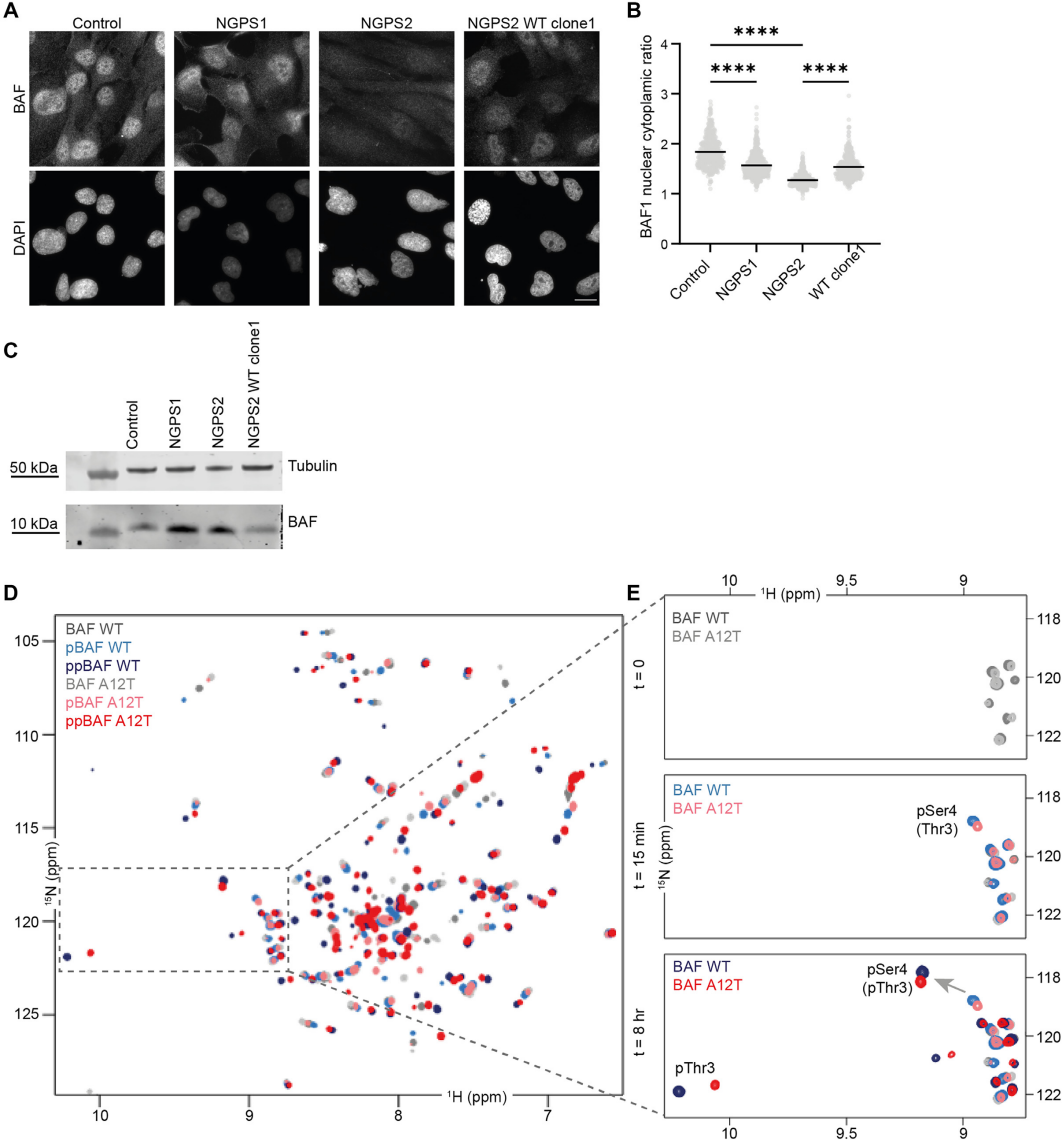
The BAF A12T mutation causes BAF mislocalization in NGPS patient cells without affecting its phosphorylation by VRK1 *in vitro*. (**A**) Representative immunofluorescence images showing endogenous BAF staining in Control, NGPS patient cells and NGPS2 WT clone1; scale bar: 20 μm. (**B**) Quantification of the mean BAF nuclear to cytoplasmic ratio in 425, 495, 401 and 363 cells for Control, NGPS1, NGPS2 and NGPS2 WTclone1, respectively. Data were obtained from three independent experiments and quantified using a one-way ANOVA analysis with Šídák’s multiple comparisons test (****P<0.0001). (**C**) Representative immunoblot showing the expression level of BAF in whole cell lysates from the indicated cell lines. Tubulin was used as a loading control. (**D–E**) 2D NMR ^1^H-^15^N Heteronuclear Single Quantum Coherence (HSQC) spectra were recorded on purified BAF WT (in shades of blue) and A12T (in shades of red) upon phosphorylation by the VRK1 kinase *in vitro*. Six spectra are superimposed, corresponding to: non-phosphorylated BAF WT and A12T (*t* = 0 min), in dark and light grey respectively, mono-phosphorylated BAF WT and A12T (*t* = 15 min), in light blue and pink respectively, and di-phosphorylated BAF WT and A12T (*t* = 8 h), in dark blue and red, respectively. The zoom in (**E**) shows the spectral regions of the ^1^H-^15^N HSQC of BAF WT and A12T spectra where signals of phosphorylated serines and threonines are commonly observed. The signals of the two BAF residues phosphorylated by VRK1 (Ser4 and Thr3) are annotated, and the arrow indicates the shift of the peak corresponding to phosphorylated Ser4 upon phosphorylation of Thr3.

In addition to the previously described delocalization of emerin from the nucleus to the cytoplasm (Figure 2C, D), we found that both NGPS cell lines showed a decrease in nuclear BAF enrichment compared to control cells, as observed by immunofluorescence, being down to 69% in the NGPS2 cells and rescued to 84% by the mutation reversion (NGPS2 WT clone 1) (Figure 3A, B). This suggested that BAF levels might be decreased in NGPS patient cells. However, immunoblot analysis instead showed that BAF levels are increased in both NGPS patient cell lines, going down upon mutation reversion (Figure 3C). Phosphorylation of BAF is known to affect its subcellular localization and to regulate its interaction with different protein partners. The decrease of nuclear BAF we observed in NGPS patient cells could thus suggest altered BAF phosphorylation upon A12T mutation. Since BAF is phosphorylated by the vaccinia-related kinase 1 (VRK1) on Serine 4 (Ser4) and then Threonine 3 (Thr3) (62,63), both being close to the mutated A12T site, we speculated that phosphorylation of BAF could be affected by the NGPS mutation.

### The A12T mutation does not affect BAF phosphorylation *in vitro*

We therefore monitored the phosphorylation of BAF WT and BAF A12T *in vitro* over time using nuclear magnetic resonance (NMR) spectroscopy (Figure 3D–E and Supplementary Figure S4). On the recorded ^1^H-^15^N HSQC NMR spectra, each peak reports on the chemical environment of one residue. A change of this residue’s chemical environment, due to a phosphorylation for example, will change the position of the corresponding NMR peak on the spectrum. Peaks of phosphorylated serine and threonine appear on the very left side of the spectrum: in general, at >9 ppm in the hydrogen dimension. Therefore, we focused our analysis on this area, where very little difference was observed between BAF WT and BAF A12T before VRK1 kinase addition (Figure 3E, top panel). After 15 min of *in vitro* phosphorylation reaction of BAF by VRK1, the signal corresponding to phosphorylated Ser4 was visible on both spectra (Figure 3E, middle panel). Similarly, after 8 h of phosphorylation, the signal corresponding to the phosphorylated Thr3 was visible on both spectra (Figure 3E, bottom panel). This phosphorylation caused a global change in both spectra (Supplementary Figure S4). Finally, no signal corresponding to another phosphorylated threonine appeared on the BAF A12T spectrum at the end of the reaction, showing that the introduced Thr12 is not phosphorylated by VRK1.

Altogether, this analysis showed that BAF WT and BAF A12T are phosphorylated in a similar way by VRK1 *in vitro*: they are both phosphorylated on Ser4 and Thr3 only, with similar kinetics, and phosphorylation of Thr3 triggers a similar global change in their ^1^H-^15^N HSQC NMR spectra, reflecting the compaction of BAF 3D structure upon phosphorylation (62).

### BAF A12T binds to lamin A/C with a ten-fold weaker affinity

**Figure 4.**
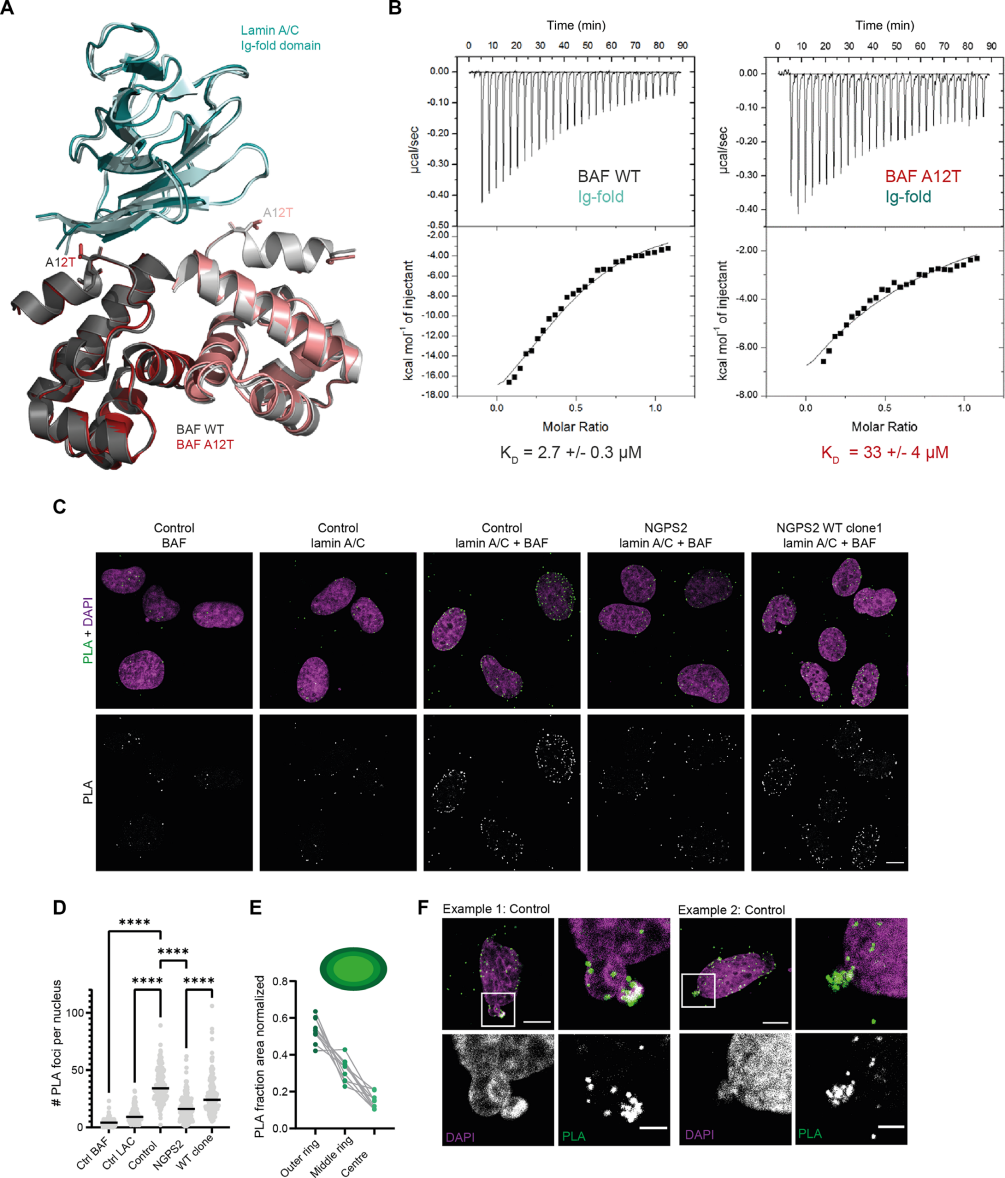
The BAF A12T mutation disrupts BAF binding to lamin A/C *in vitro* and in NGPS cells. (**A**) Superimposition of the 3D structure of BAF A12T (in light pink) bound to the lamin A/C Ig-fold domain (in light blue), on to the previously reported structure of BAF WT bound to this same lamin A/C domain (in grey; PDB code: 6GHD). Binding free energies calculated from these 3D structures are indicated in Supplementary Figure S5a. (**B**) ITC curves reporting binding of BAF (either WT or A12T) to the lamin A/C Ig-fold domain. The experiments were performed twice and the mean dissociation constant (*K*_D_) values are shown under one representative curve. The duplicated experiments are shown in Supplementary Figure S5b. The thermodynamic parameters deduced from all experiments are summarized in Supplementary Figure S5c. (**C**) Representative confocal images of the signal obtained by PLA using either BAF or lamin A/C antibodies alone as negative controls or as a combination in the indicated cell lines. The top row shows merged pictures of the PLA signal (green) and DAPI (magenta); scale bar: 10 μm. (**D**) Quantification of the number of PLA foci per nucleus. The data represent the mean PLA signal measured in individual nuclei in *n* = 214 (Control BAF), 187 (Control lamin A/C), 229 (Control lamin A/C+BAF), 213 (NGPS2 lamin A/C +BAF) and 219 cells (NGPS2 WT clone1, lamin A/C + BAF). One-way ANOVA analysis with Šídák’s multiple comparisons test (****P<0.0001). (**E**) Analysis of the nuclear distribution of PLA foci in the middle slice of a z-stack obtained from confocal images in control cells using CellProfiler. The nucleus was subdivided in concentric circles going from the nuclear periphery to the nuclear interior and the fraction of PLA foci in each of these circles was counted per nucleus (*n* = 10 nuclei). (**F**) Example confocal images (from images as shown in C) showing the BAF-lamin A/C PLA signal accumulation at nuclear bleb sites in Control cells. Scale bar: 10 and 3 μm (zoom).

In order to further characterize the effect of the A12T mutation on BAF 3D structure and binding properties, we initiated a structural analysis of BAF A12T by X-ray crystallography. The only interaction known to directly involve alanine 12 is the interaction of BAF with the Ig-fold domain of lamin A/C (32). Therefore, we determined the 3D structure of BAF A12T bound to the lamin A/C Ig-fold domain at 1.6 Å resolution (Figure 4A). We compared the structure of this complex to that of the wild-type complex (PDB code: 6GHD) and found that they nicely overlapped (CαRMSD = 0.4 Å) with Ala12 or Thr12 being largely exposed to the solvent (Figure 4A). Only slight changes in the relative positioning of the BAF monomers, as well as in the structure of some lamin A/C loops (especially at Ser433-Thr434) were observed. We thus concluded that the A12T mutation does not affect BAF 3D structure. It has been proposed that the A12T mutation perturbs the interaction with the Ig-fold domain of lamin A/C (32). Additionally, this interaction is important for the retention of BAF in the nucleus (64,65).

We investigated the effect of the BAF A12T mutation on the binding affinity to the lamin A/C Ig-fold domain *in vitro*. Despite an apparent similarity between the 3D structures of the WT and mutated complexes, prediction of the binding energies from these 3D structures using the PDBePISA server (66) suggested that the A12T mutation changes the thermodynamical equilibrium of the system, with the mutant binding with a free energy Δ*G* that is 1.8 kcal/mol less favourable compared to the WT (Supplementary Figure S5a). To confirm this prediction experimentally, we first showed by NMR that the A12T mutation causes a significant decrease in BAF affinity for lamin A/C Ig-fold domain (Supplementary Figure S5d–k). Therefore, we measured the intensities of the ^1^H-^15^N HSQC peaks of the Ig-fold domain in the presence of different ratios of BAF WT and BAF A12T. After binding to BAF WT dimer at 1:1 and 1:2 ratio, only 27% and 4% of the initial NMR signals were observed, respectively (in light and dark blue in Supplementary Figure S5f, h, k). In the case of BAF A12T dimer, using the same ratios (1:1 and 1:2), we measured lower intensity decreases: 43 and 66% respectively (in pink and red in Supplementary Figure S5g, i, k). This indicated that significantly less complexes are formed when BAF alanine 12 is mutated into threonine. We then determined the dissociation constant (*K*_D_) of these interactions using Isothermal Titration Calorimetry (ITC). As the affinities were on the (high) micromolar range, the solubility limits of our proteins prevented reaching saturation. To improve the quality of our data analysis, we decided to fit our ITC curves assuming a stoichiometry of two BAF molecules for one lamin A/C Ig-fold domain. Indeed, we already reported that BAF A12T eluted from the size-exclusion chromatography column at the same volume as BAF WT, which demonstrates that BAF A12T is a dimer in solution (32). Also, both BAF WT and A12T dimers bind to a single Ig-fold domain in the crystal structures. We obtained *K*_D_ values of 2.7 ± 1.2 μM and 33 ± 4 μM for the interactions involving BAF WT and BAF A12T, respectively (Figure 4B and Supplementary Figure S5b, c), showing that the A12T mutation decreases the affinity of BAF for lamin A/C Ig-fold by about tenfold.

To assess whether the reduced interaction between BAF A12T and the lamin A/C Ig-fold *in vitro* was also translated in a cellular context, we used a PLA. While each of the BAF or lamin A/C antibodies on their own gave very little PLA signal as expected, specific PLA foci were detected upon combining the two antibodies, confirming the proximity of the two proteins in control cells. Furthermore, we observed a strong reduction in the number of PLA foci in NGPS2 cells, reflecting a decreased interaction between BAF-lamin A/C, which was rescued by reversion of the mutation (Figure 4C, D). Even though BAF and lamin A/C are both found throughout the nucleus, we observed a clear enrichment of PLA foci at the nuclear periphery. Indeed, by measuring the number of PLA foci in concentric rings within the nucleus, starting from the nuclear periphery, we confirmed that most of the PLA foci were present in the outer nuclear ring (Figure 4E), indicating that these proteins mostly interact at the NE. In addition, we noticed enrichment of PLA foci in nuclear blebs of a subset of control cells, indicating that blebs could be a site of increased interaction between BAF and lamin A/C (Figure 4F). Together these data show that the BAF A12T mutation reduces the affinity of BAF for lamin A/C both *in vitro* and in NGPS cells.

### Reduced binding of BAF A12T to lamin A/C in NGPS cells prevents lamin A/C recruitment to NE ruptures

**Figure 5.**
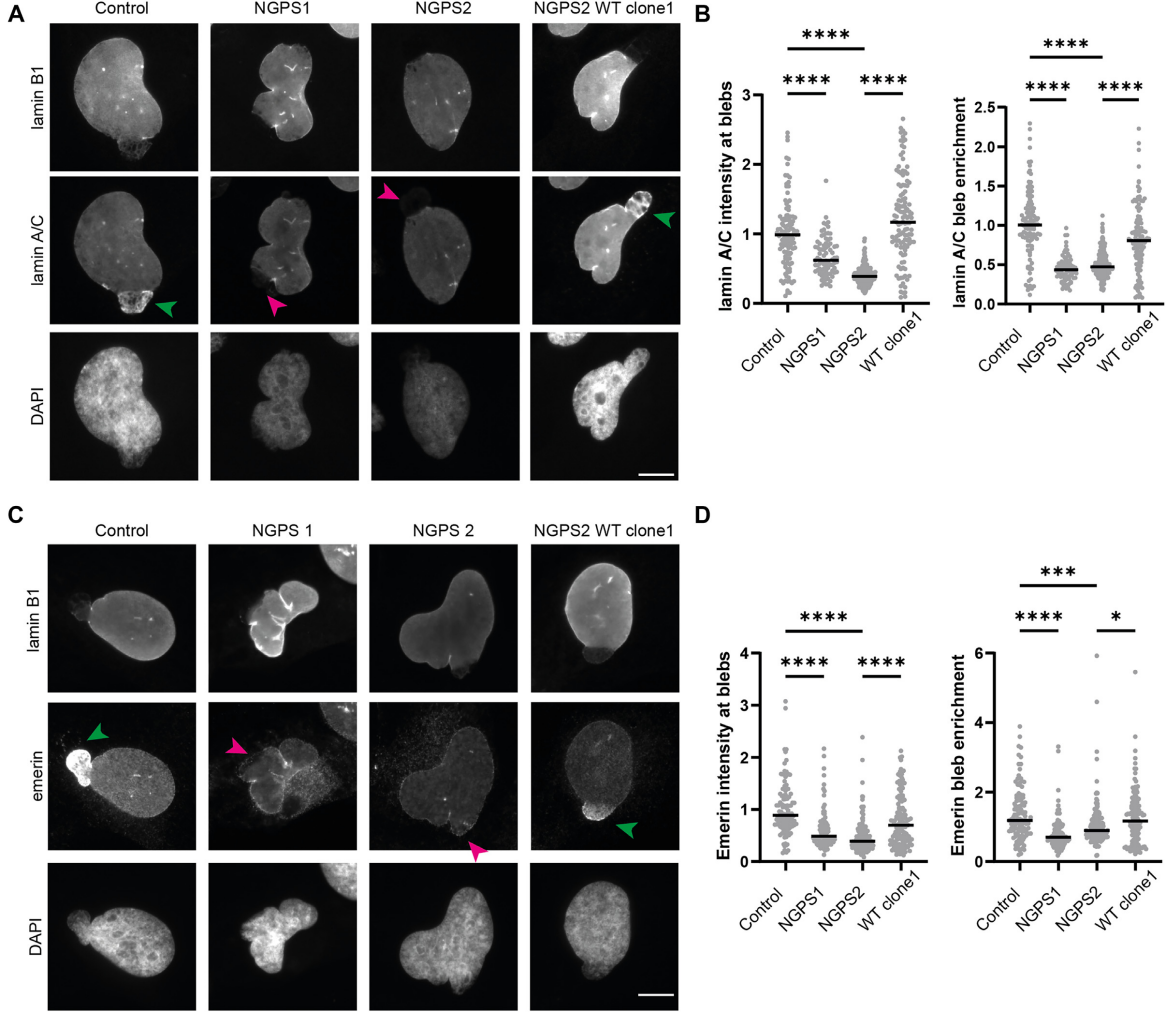
The BAF A12T mutation in NGPS patient cells prevents the recruitment of lamin A/C and emerin to nuclear blebs. (**A**) Representative immunofluorescence images of endogenous lamin B1, and lamin A/C at nuclear blebs in Control, NGPS patient cells and NGPS2 WT clone1 cells. Arrows indicate the enrichment (green) or lack of (magenta) lamin A/C at blebs; scale bar: 10 μm. (**B**) Quantification of the normalized lamin A/C intensity at blebs and of the enrichment of lamin A/C at nuclear bleb regions compared to the levels measured in the rest of the nucleus. Blebs were identified by the lack of lamin B1 staining. Data points represent 117 (Control), 81 (NGPS1), 235 (NGPS2), and 122 (WT clone1) individual blebs from three independent experiments, the median is indicated. One-way ANOVA analysis with Šídák’s multiple comparisons test (****P<0.0001). (**C**) Representative immunofluorescence images of lamin B1 and emerin in nuclear blebs of Control, NGPS patient cells and NGPS2 WT clone1 cells. Arrows indicate the enrichment (green) or lack of (magenta) emerin at blebs; scale bar: 10 μm. (**D**) Quantification of the normalized emerin intensity at blebs and the enrichment of emerin at bleb regions compared to levels in the rest of the nucleus. Data points represent 113 (Control), 87 (NGPS1), 163 (NGPS2) and 134 (WT clone1) individual blebs from three independent experiments, and the median is indicated. The data were analysed with a one-way ANOVA analysis with Šídák’s multiple comparisons test (****P<0.0001, ***P<0.005, *P<0.05).

Recruitment of lamin A/C by BAF at sites of nuclear ruptures has recently been shown to be an early event in the repair of nuclear envelope ruptures and ultimately leaves behind a lamin ‘scar’ (65,67). We observed these lamin scars at the NE of control cells (Figure 1A, green arrowheads) but in contrast, often observed gaps in the lamina of NGPS patient cells (Figure 1A, magenta arrowheads). Gaps in the lamina can generate weak spots in the NE leading to bleb formation and ultimately NE rupture. We therefore investigated the effect of the BAF A12T mutation on the recruitment of lamin A/C to nuclear blebs, which can be recognized on immunofluorescence images by a lack of B-type lamins (20). In control cells, we observed a clear accumulation of lamin A/C to a subpopulation of blebs (Figure 5A, green arrowheads). NGPS cells on the other hand did not show lamin A/C recruitment to blebs (Figure 5A, magenta arrowheads). Automated quantification using a CellProfiler based pipeline (45) of either lamin A/C intensity at blebs or enrichment of lamin A/C at blebs, confirmed a lack of lamin A/C recruitment in NGPS cells (Figure 5B). Reversion of the BAF A12T mutation in NGPS2 cells was able to fully rescue the lamin A/C recruitment defect (Figure 5A [green arrowheads] and 5b, Supplementary Figure S6a, b), demonstrating that this phenotype is caused by the mutation. Additionally, we examined the recruitment of emerin to blebs, as emerin is also known to accumulate at rupture sites in the NE repair process and because emerin localization is altered in NGPS patient cells. We found a decrease in emerin intensity at NE blebs in NGPS cells, that was fully rescued by the mutation reversion, which could be partially explained by an overall decrease in emerin levels at the NE (Figure 5C, D, Supplementary Figure S6c, d).

### Wild-type BAF overexpression in NGPS cells is sufficient to restore lamin A/C recruitment to NE ruptures

**Figure 6.**
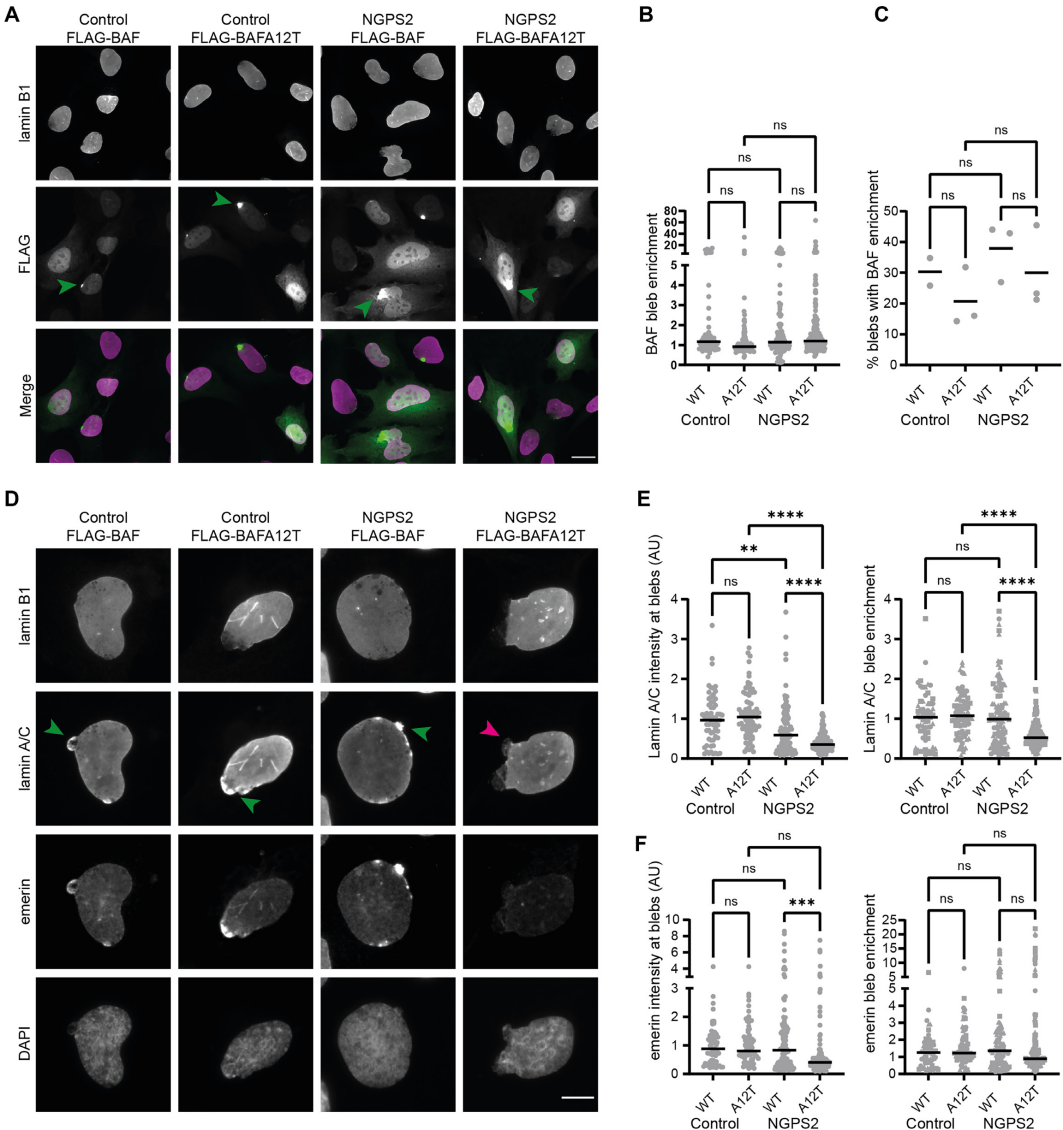
The defective recruitment of lamin A/C to nuclear blebs is restored in NGPS cells by BAF wild-type overexpression. (**A**) Representative immunofluorescence images of FLAG and lamin B1 staining in Control and NGPS2 cells stably expressing FLAG-BAF WT or A12T. Green arrows indicate FLAG-BAF accumulation at nuclear blebs. Scale bar: 20 μm. (**B**) Quantification of FLAG-BAF WT and A12T enrichment at bleb regions compared to the levels measured in the rest of the nucleus. Blebs were identified by the lack of lamin B1 staining. Data points represent *n* = 56 (Control FLAG-BAF), 82 (Control FLAG-BAFA12T), 112 (NGPS2 FLAG-BAF) and 156 (NGPS2 FLAG-BAFA12T) individual blebs from three independent experiments, and median is indicated. One-way ANOVA analysis with Šídák’s multiple comparisons test showed no significant (ns) differences. (**C**) For each experiment in (B) the percentage of cells showing >1.5 FLAG-BAF enrichment at blebs was calculated. Mixed-effects analysis with Šídák’s multiple comparisons test showed no significant (ns) differences, and mean is indicated. (**D**) Representative immunofluorescence images of lamin B1, lamin A/C and emerin staining in Control and NGPS2 cells stably expressing FLAG-BAF WT or A12T. Arrows point to nuclear blebs with accumulation (green arrowheads) or lack of (magenta arrowheads) lamin A/C. Scale bar: 10 μm. (**E**) Quantification of the normalized lamin A/C intensity at blebs and of the enrichment of lamin A/C at bleb regions compared to the levels measured in the rest of the nucleus. Blebs were identified by the lack of lamin B1 staining. Data points represent *n* = 61 (Control FLAG-BAF), 79 (Control FLAG-BAFA12T), 108 (NGPS2 FLAG-BAF) and 153 (NGPS2 FLAG-BAFA12T) individual blebs from three independent experiments, median is indicated. One-way ANOVA analysis with Šídák’s multiple comparisons test (****P<0.0001, **P<0.01). (**F**) Quantification of the normalized emerin intensity at blebs and of the enrichment of emerin at bleb regions compared to the levels measured in the rest of the nucleus. Data points represent *n* = 61 (Control FLAG-BAF), 79 (Control FLAG-BAFA12T), 108 (NGPS2 FLAG-BAF) and 153 (NGPS2 FLAG-BAFA12T) individual blebs from three independent experiments. One-way ANOVA analysis with Šídák's multiple comparisons test (***P<0.001).

To assess whether overexpression of wild-type BAF in NGPS cells could rescue the observed defects in the recruitment of lamin A/C or emerin to blebs, we generated control and NGPS patient cells stably expressing FLAG-BAF wild-type (WT) or FLAG-BAF A12T. This mimics a situation where BAF A12T is present as a heterozygous mutation, as it occurs in NGPS patients’ parents, who are devoid of any disease phenotype. First, we assessed FLAG-BAF (WT and A12T) localization in these cells and observed that both proteins localized to the nucleus as expected (Figure 6A). We additionally checked whether BAF A12T recruitment to rupture sites was affected, as contradictory results on the affinity of BAF A12T for DNA were previously published (62,68). We observed that both FLAG-BAF WT and A12T localized to blebs identified by lack of lamin B in both Control and NGPS2 background (Figure 6A, B). Around 30% of all blebs showed FLAG-BAF enrichment for both the WT and the A12T protein (Figure 6C). Together, these results suggested that the recruitment of BAF to DNA at nuclear rupture sites is not affected by the A12T mutation, consistent with *in vitro* data (62) and with recent reports by others in cells (67). In addition, we observed that emerin nuclear localization was rescued by overexpression of FLAG-BAF WT in NGPS patient cells (Supplementary Figure S7a, b). Finally, we showed that overexpression of FLAG-BAF WT in NGPS patient cells was sufficient to rescue lamin A/C and emerin recruitment to NE blebs (Figure 6D–F). Similarly, overexpression of BAF A12T in a wild-type background did not cause defects in the recruitment of these proteins. Thus the presence of wild-type BAF prevents or reverses the observed phenotypes which is consistent with the lack of disease phenotypes in NGPS parents who carry a BAF A12T heterozygous mutation (39,40).

**Figure 7.**
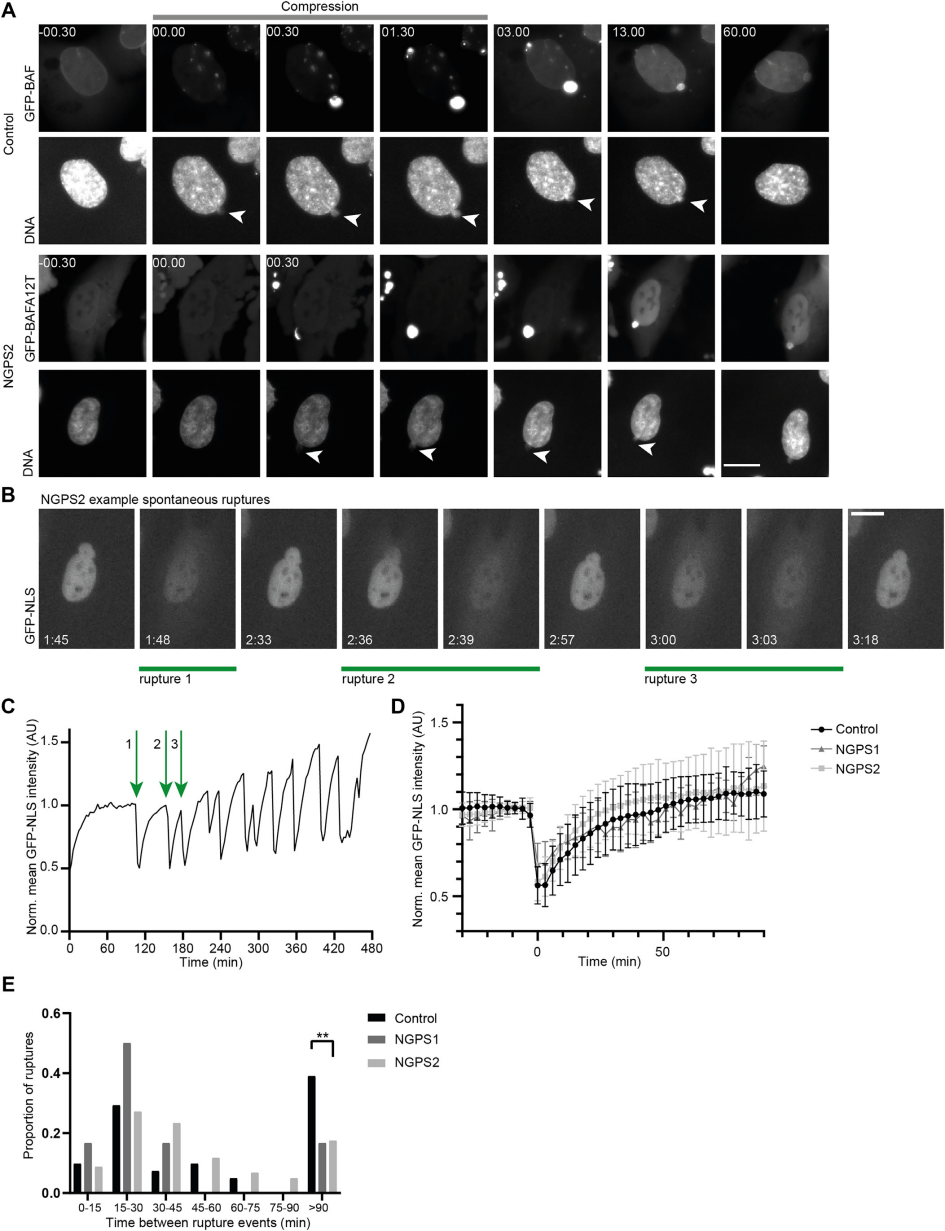
The NE rupture repair kinetic is unaffected in NGPS cells but nuclei are more prone to re-rupturing. (**A**) Still images of Control GFP-BAF WT and NGPS2 GFP-BAF A12T cells showing nuclear rupture upon compression (white arrowheads). Time is indicated in min:sec relative to the first time point of compression; scale bar: 20 μm. (**B**) Example of still images from a time lapse obtained in NGPS2 cells stably expressing NLS-GFP and showing several rupture and repair events originating from the same nuclear bleb. Time is indicated in hr:min; scale bar: 20 μm. (**C**) Graph showing the mean GFP-NLS fluorescence intensity over time in the nucleus shown in (B). The repetitive ruptures depicted in (A) are indicated on the graph by green arrows. (**D**) Analysis of the recovery kinetics of nuclear GFP-NLS after major NE rupture events (>30% loss of nuclear GFP-NLS fluorescence intensity). Mean and standard deviation is indicated for control (black, *n* = 62 ruptures), NGPS1 (dark grey, *n* = 7 ruptures), NGPS2 (light grey, *n* = 110 ruptures). (**E**) Histogram showing the proportion of ruptures with the indicated duration between rupture events after an initial major rupture occurred. Control (black, *n* = 41 ruptures), NGPS1 (dark grey, *n* = 6 ruptures) and NGPS2 (light grey, *n* = 103 ruptures) cells were imaged every 3 min and were binned to every 15 min. Fisher’s exact test (**P<0.01).

Since we identified that BAF A12T accumulates at NE ruptures in a similar way as BAF WT, we next sought to investigate the dynamics of BAF recruitment to these ruptures. Therefore, we performed live cell imaging on control cells stably expressing GFP-BAF WT and on NGPS2 cells stably expressing GFP-BAF A12T. To study the dynamics with a high temporal resolution, nuclear rupture was induced by cellular compression. Both GFP-BAF WT and GFP-BAF A12T were rapidly recruited to rupture sites (Figure 7A) after compression. Recruitment to chromatin herniations (white arrowheads) occurred within 30 s. After release of cellular compression, GFP-BAF A12T in NGPS2 and GFP-BAF WT in control cells dissociated from the site of rupture at a similar rate. Therefore, these data suggest that the recruitment to and persistence of BAF at sites of ruptures is not affected by the A12T mutation.

### The reduced accumulation of lamin A/C to NE ruptures in NGPS cells does not affect NE rupture repair but causes NE re-rupturing

The specific role of lamin A/C in nuclear envelope rupture repair is yet to be identified. No associated defect in NE repair and integrity has been reported yet as a consequence of a lack of lamin A/C recruitment to sites of nuclear rupture. We, therefore, monitored NE rupture and repair kinetics following spontaneous rupture events by imaging of stably expressed fluorescently tagged nuclear localization signal (GFP-NLS) in control and NGPS cells. Upon NE rupture, the GFP-NLS reporter leaks into the cytoplasm. Once the rupture has been repaired, GFP-NLS reaccumulates into the nucleus. Using live cell imaging of GFP-NLS, we observed spontaneous ruptures in all cell lines. Some cells showed repetitive NE rupture events during the time of observation, which was surprisingly not associated with cell death and appeared to be repaired quickly (Figure 7B, C). As BAF-dependent NE repair was proposed to be more important for large membrane gaps (31), we decided to focus our analysis on major NE rupture events, that we defined as being the ones leading to loss of >30% of the mean nuclear GFP-NLS intensity. We found that the lack of lamin A/C recruitment did not interfere with the repair process as we did not find a delay in the repair kinetics when comparing both NGPS cell lines to the control (Figure 7D).

We then analysed the re-occurrence of ruptures after an initial major rupture event and observed that both NGPS cell lines were more likely to re-rupture within 90 min compared to control cells (Figure 7E). These data showed the same trend for both NGPS cell lines, statistical analysis did however not find a significant difference for NGPS1 compared to the control, probably due to the low number of recorded ruptures in this cell line overall. Altogether, our data show that the lack of lamin A/C recruitment to sites of NE rupture causes NE instability through recurrent rupturing of the NE. This is associated with the persistence of a lamina gap after initial NE repair has occurred.

## DISCUSSION

In the last ten years, the interest in the small DNA binding protein BAF has grown due to two main discoveries. First, a recessive mutation in BAF, namely A12T, causes Nestor–Guillermo Progeria Syndrome. Second, BAF plays a role in the repair of nuclear envelope ruptures. BAF directly binds to two well-characterized nuclear envelope proteins: A-type lamins and emerin. A recent report showed that, in fibroblasts overexpressing GFP-BAF A12T, recruitment of A-type lamins to laser-induced nuclear ruptures is impaired (67). However, until now, the mechanism behind this phenotype was unknown. Here, we revealed a mechanism for the defective recruitment of A-type lamins at the nuclear envelope ruptures and we further characterized the consequences of this disease-associated mechanism on the nuclear envelope integrity in the context of NGPS cells.

Using a combination of *in vitro* and cell-based experiments, we showed that, while the A12T mutation did not affect BAF 3D structure nor its phosphorylation by VRK1, it significantly reduces its binding affinity for the Igfold domain of lamin A/C. As a consequence, we found that the accumulation of A-type lamins at nuclear rupture sites—known to be dependent on the presence of BAF (31)—was strongly reduced in NGPS patient cells. We confirmed that this was indeed a specific effect of the BAF mutation by correcting the BAF A12T homozygous mutation using CRISPR-Cas9. We observed that the A12T mutation also causes delocalization of emerin from the NE to the cytoplasm in NGPS cells, even though this mutation is not expected to impair BAF-emerin binding directly. Both BAF and lamin are important for the nuclear enrichment and dynamics of emerin (69,70). Therefore, we speculate that inefficient immobilization of emerin in BAF-emerin-lamin A/C tertiary complexes at the NE caused by the reduced binding of BAF to lamin A/C could affect emerin nuclear localization. This in turn could have additional effects on downstream emerin functions such as mechanotransduction processes at the NE. Interestingly, NGPS cells show an increase in BAF levels which could be a mechanism to compensate for a decrease in BAF–lamin A/C interaction and a defect in associated cellular processes.

While BAF is important for recruiting A-type lamins to NE ruptures, the presence of lamin A has been shown to be reciprocally important for the accumulation of BAF in the nucleus (65,69) and A-type lamins have been suggested to be involved in BAF accumulation at rupture sites (65). However, we and others (67) did not find an obvious defect in BAF A12T recruitment to sites of nuclear ruptures. In addition, we did not find any change in the NE repair kinetics between NGPS and control cells. As BAF A12T can still localize to the exposed DNA at the rupture site, it is likely to still be able to recruit downstream proteins required for the repair process, especially as the mutation is not predicted to interfere with LEM domain protein interactions. We did observe a reduction in emerin recruitment to NE ruptures even though this did not seem to interfere with the repair process. We propose that either multiple LEM domain proteins contribute to NE repair or the partial recruitment of emerin in NGPS cells is sufficient for its repair function at the NE. We also showed that overexpression of FLAG-BAF WT in NGPS patient cells was sufficient to rescue lamin A/C and emerin recruitment, and overexpression of BAF A12T in a wild-type background did not cause defects in the recruitment of these proteins, which explains the lack of disease phenotypes in NGPS parents who carry a BAF A12T heterozygous mutation (39,40).

Finally, our results revealed that the NE of NGPS cells was more likely to re-rupture after an initial rupture event, supporting a role for A-type lamins in strengthening the ruptured NE. So far, it was hypothesized that A-type lamin recruitment to sites of NE ruptures stabilize the nuclear envelope after it had ruptured, thereby protecting its integrity (20). However, experimental evidence for the role of A-type lamins recruitment by BAF at the NE was lacking. Here, we demonstrated that, in the presence of BAF A12T, lack of A-type lamins accumulation at sites of ruptures does not affect the dynamics of NE repair but leaves areas of the NE devoid of lamin A/C that are more prone to re-rupture. Our data suggest that the role of the BAF–lamin A/C interaction is to increase the robustness of the NE at the sites of rupture. Several mutations in the Ig-fold domain of lamin A/C found in progeroid laminopathies have recently been shown to impair binding to BAF (32), leading to reduced recruitment of A-type lamins to rupture sites (65,67), with unknown consequences. This suggests a common mechanism for these diseases, in which lack of A-type lamins at the rupture sites creates fragile sites in the NE that are more prone to re-rupture. This mechanism could contribute to the tissue-specific severity observed in progerias and in other laminopathies. Indeed, the most severely affected tissues are the ones subjected to higher mechanical stress (heart and vascular tissues in HGPS) and/or very dense tissues (bone tissues in NGPS patients). These tissues are therefore likely to be more prone to NE ruptures.

Altogether, our work, as well as these recent studies, suggest that, once exposed to continuous mechanical forces and therefore more frequent re-ruptures, NGPS cells might over time accumulate DNA damage and/or activate inflammatory pathways that could contribute to the premature aging disease pathology.

## DATA AVAILABILITY

Structure coordinates were deposited to the PDB, with entry 7Z21.

## Supplementary Material

gkac726_Supplemental_File

## References

[1] Taddei A. , Hediger F., Neumann F.R., Gasser S.M. The function of nuclear architecture: a genetic approach. Annu. Rev. Genet. 2004; 38:305–345. 15568979 10.1146/annurev.genet.37.110801.142705

[2] Dechat T. , Adam S.A., Goldman R.D. Nuclear lamins and chromatin: when structure meets function. Adv Enzyme Regul. 2009; 49:157. 19154754 10.1016/j.advenzreg.2008.12.003 PMC3253622

[3] Crisp M. , Liu Q., Roux K., Rattner J.B., Shanahan C., Burke B., Stahl P.D., Hodzic D. Coupling of the nucleus and cytoplasm: role of the LINC complex. J. Cell Biol. 2006; 172:41–53. 16380439 10.1083/jcb.200509124 PMC2063530

[4] Lombardi M.L. , Jaalouk D.E., Shanahan C.M., Burke B., Roux K.J., Lammerding J. The interaction between nesprins and sun proteins at the nuclear envelope is critical for force transmission between the nucleus and cytoskeleton. J. Biol. Chem. 2011; 286:26743–26753. 21652697 10.1074/jbc.M111.233700 PMC3143636

[5] Gruenbaum Y. , Margalit A., Goldman R.D., Shumaker D.K., Wilson K.L. The nuclear lamina comes of age. Nat. Rev. Mol. Cell Biol. 2005; 6:21–31. 15688064 10.1038/nrm1550

[6] Dechat T. , Pfleghaar K., Sengupta K., Shimi T., Shumaker D.K., Solimando L., Goldman R.D. Nuclear lamins: major factors in the structural organization and function of the nucleus and chromatin. Genes Dev. 2008; 22:832–853. 18381888 10.1101/gad.1652708 PMC2732390

[7] Vahabikashi A. , Adam S.A., Medalia O., Goldman R.D. Nuclear lamins: structure and function in mechanobiology. APL Bioeng. 2022; 6:11503. 10.1063/5.0082656 PMC8810204 35146235

[8] Worman H.J. , Bonne G. Laminopathies”: a wide spectrum of human diseases. Exp. Cell. Res. 2007; 313:2121–2133. 17467691 10.1016/j.yexcr.2007.03.028 PMC2964355

[9] Cohen T. V , Hernandez L., Stewart C.L. Functions of the nuclear envelope and lamina in development and disease. Biochem. Soc. Trans. 2008; 36:1329–1334. 19021550 10.1042/BST0361329

[10] Goldman R.D. , Gruenbaum Y., Moir R.D., Shumaker D.K., Spann T.P. Nuclear lamins: building blocks of nuclear architecture. Genes Dev. 2002; 16:533–547. 11877373 10.1101/gad.960502

[11] De Sandre-Giovannoli A. , Bernard R., Cau P., Navarro C., Amiel J., Boccaccio I., Lyonnet S., Stewart C.L., Munnich A., Le Merrer M. et al. Lamin a truncation in Hutchinson-Gilford progeria. Science. 2003; 300:2055. 12702809 10.1126/science.1084125

[12] De Sandre-Giovannoli A. , Chaouch M., Kozlov S., Vallat J.M., Tazir M., Kassouri N., Szepetowski P., Hammadouche T., Vandenberghe A., Stewart C.L. et al. Homozygous defects in LMNA, encoding lamin A/C nuclear-envelope proteins, cause autosomal recessive axonal neuropathy in human (Charcot-Marie-Tooth disorder type 2) and mouse. Am. J. Hum. Genet. 2002; 70:726–736. 11799477 10.1086/339274 PMC384949

[13] De Vos W.H. , Houben F., Kamps M., Malhas A., Verheyen F., Cox J., Manders E.M.M., Verstraeten V.L.R.M., Van steensel M.A.M., Marcelis C.L.M. et al. Repetitive disruptions of the nuclear envelope invoke temporary loss of cellular compartmentalization in laminopathies. Hum. Mol. Genet. 2011; 20:4175–4186. 21831885 10.1093/hmg/ddr344

[14] Muchir A. , Medioni J., Laluc M., Massart C., Arimura T., Van Der Kooi A.J., Desguerre I., Mayer M., Ferrer X., Briault S Nuclear envelope alterations in fibroblasts from patients with muscular dystrophy, cardiomyopathy, and partial lipodystrophy carrying lamin A/C gene mutations. Muscle Nerve. 2004; 30:444–450. 15372542 10.1002/mus.20122

[15] Kim P.H. , Chen N.Y., Heizer P.J., Tu Y., Weston T.A., Fong J.L.C, Gill N.K., Rowat A.C., Young S.G., Fong L.G. Nuclear membrane ruptures underlie the vascular pathology in a mouse model of Hutchinson-Gilford progeria syndrome. JCI Insight. 2021; 6:e151515. 10.1172/jci.insight.151515 PMC8409987 34423791

[16] Cohen S. , Marr A.K., Garcin P., Panté N. Nuclear envelope disruption involving host caspases plays a role in the parvovirus replication cycle. J. Virol. 2011; 85:4863–4874. 21367902 10.1128/JVI.01999-10 PMC3126209

[17] de Noronha C.M. , Sherman M.P., Lin H.W., Cavrois M. V, Moir R.D., Goldman R.D., Greene W.C. Dynamic disruptions in nuclear envelope architecture and integrity induced by HIV-1 Vpr. Science (80-.). 2001; 294:1105–1108. 10.1126/science.1063957 11691994

[18] Earle A.J. , Kirby T.J., Fedorchak G.R., Isermann P., Patel J., Iruvanti S., Moore S.A., Bonne G., Wallrath L.L., Lammerding J. Mutant lamins cause nuclear envelope rupture and DNA damage in skeletal muscle cells. Nat. Mater. 2020; 19:464–473. 31844279 10.1038/s41563-019-0563-5 PMC7102937

[19] Raab M. , Gentili M., De Belly H., Thiam H.R., Vargas P., Jimenez A.J., Lautenschlaeger F., Voituriez R., Lennon-Duménil A.M., Manel N. et al. ESCRT III repairs nuclear envelope ruptures during cell migration to limit DNA damage and cell death. Science (80-.). 2016; 352:359–362. 10.1126/science.aad7611 27013426

[20] Denais C.M. , Gilbert R.M., Isermann P., McGregor A.L., Lindert M., Weigelin B., Davidson P.M., Friedl P., Wolf K., Lammerding J. Nuclear envelope rupture and repair during cancer cell migration. Science (80-.). 2016; 352:353–358. 10.1126/science.aad7297 PMC4833568 27013428

[21] Xia Y. , Ivanovska I.L., Zhu K., Smith L., Irianto J., Pfeifer C.R., Alvey C.M., Ji J., Liu D., Cho S. et al. Nuclear rupture at sites of high curvature compromises retention of DNA repair factors. J. Cell Biol. 2018; 217:3796–3808. 30171044 10.1083/jcb.201711161 PMC6219729

[22] Tamiello C. , Kamps M.A.F., Van den Wijngaard A., Verstraeten V.L.R.M., Baaijens F.P.T., Broers J.L.V., Bouten C.C.V. Soft substrates normalize nuclear morphology and prevent nuclear rupture in fibroblasts from a laminopathy patient with compound heterozygous LMNA mutations. Nucleus. 2013; 4:61–73. 23324461 10.4161/nucl.23388 PMC3585029

[23] Hatch E. , Hetzer M. Breaching the nuclear envelope in development and disease. J. Cell Biol. 2014; 205:133–141. 24751535 10.1083/jcb.201402003 PMC4003239

[24] Le Berre M. , Aubertin J., Piel M. Fine control of nuclear confinement identifies a threshold deformation leading to lamina rupture and induction of specific genes. Integr. Biol. 2012; 4:1406–1414. 10.1039/c2ib20056b 23038068

[25] Deviri D. , Pfeifer C.R., Dooling L.J., Ivanovska I.L., Discher D.E., Safran S.A. Scaling laws indicate distinct nucleation mechanisms of holes in the nuclear lamina. Nat. Phys. 2019; 15:823–829.

[26] Pfeifer C.R. , Xia Y., Zhu K., Liu D., Irianto J., García V.M.M., Millán L.M.S., Niese B., Harding S., Deviri D. Constricted migration increases DNA damage and independently represses cell cycle. Mol. Biol. Cell. 2018; 29:1948–1962. 29742017 10.1091/mbc.E18-02-0079 PMC6232975

[27] Ablasser A. , Chen Z.J. cGAS in action: Expanding roles in immunity and inflammation. Science (80-.). 2019; 363:eaat8657. 10.1126/science.aat8657 30846571

[28] Guey B. , Wischnewski M., Decout A., Makasheva K., Kaynak M., Saka M.S., Fierz B., Ablasser A. BAF restricts cGAS on nuclear DNA to prevent innate immune activation. Science (80-.). 2020; 369:823–828. 10.1126/science.aaw6421 32792394

[29] Halfmann C.T. , Sears R.M., Katiyar A., Busselman B.W., Aman L.K., Zhang Q., Bryan C.S.O., Angelini T.E., Lele T.P., Roux K.J. Repair of nuclear ruptures requires barrier-to- autointegration factor. J. Cell Biol. 2019; 218:2136–2149. 31147383 10.1083/jcb.201901116 PMC6605789

[30] Robijns J. , Molenberghs F., Sieprath T., Corne T.D.J., Verschuuren M., De Vos W.H. In silico synchronization reveals regulators of nuclear ruptures in lamin A/C deficient model cells. Sci. Rep. 2016; 6:30325. 27461848 10.1038/srep30325 PMC4962089

[31] Young A.M. , Gunn A.L., Hatch E.M. BAF facilitates interphase nuclear membrane repair through recruitment of nuclear transmembrane proteins. Mol. Biol. Cell. 2020; 31:1551–1560. 32459568 10.1091/mbc.E20-01-0009 PMC7521799

[32] Samson C. , Petitalot A., Celli F., Herrada I., Ropars V., Le Du M.H., Nhiri N., Jacquet E., Arteni A.A., Buendia B. et al. Structural analysis of the ternary complex between lamin A/C, BAF and emerin identifies an interface disrupted in autosomal recessive progeroid diseases. Nucleic Acids Res. 2018; 46:10460–10473. 30137533 10.1093/nar/gky736 PMC6212729

[33] Cai M. , Huang Y., Suh J.-Y., Louis J.M., Ghirlando R., Craigie R., Clore G.M. Solution NMR structure of the barrier-to-autointegration factor-Emerin complex. J. Biol. Chem. 2007; 282:14525–14535. 17355960 10.1074/jbc.M700576200

[34] Bradley C.M. , Ronning D.R., Ghirlando R., Craigie R., Dyda F. Structural basis for DNA bridging by barrier-to-autointegration factor. Nat. Struct. Mol. Biol. 2005; 12:935–936. 16155580 10.1038/nsmb989

[35] Zheng R. , Ghirlando R., Lee M.S., Mizuuchi K., Krause M., Craigie R. Barrier-to-autointegration factor (BAF) bridges DNA in a discrete, higher-order nucleoprotein complex. Proc. Natl. Acad. Sci. U.S.A. 2000; 97:8997–9002. 10908652 10.1073/pnas.150240197 PMC16810

[36] Haraguchi T. , Koujin T., Segura-Totten M., Lee K.K., Matsuoka Y., Yoneda Y., Wilson K.L., Hiraoka Y. BAF is required for emerin assembly into the reforming nuclear envelope. J. Cell Sci. 2001; 114:4575–4585. 11792822 10.1242/jcs.114.24.4575

[37] de Oca R.M. , Andreassen P.R., Wilson K.L. Barrier-to-autointegration factor influences specific histone modifications. Nucleus. 2011; 2:580–590. 22127260 10.4161/nucl.2.6.17960 PMC3324346

[38] Jamin A. , Wicklund A., Wiebe M.S. Cell- and Virus-Mediated Regulation of the Barrier-to-Autointegration Factor's Phosphorylation State Controls Its DNA Binding, Dimerization, Subcellular Localization, and Antipoxviral Activity. J. Virol. 2014; 88:5342–5355. 24600006 10.1128/JVI.00427-14 PMC4019112

[39] Cabanillas R. , Cadiñanos J., Villameytide J.A.F., Pérez M., Longo J., Richard J.M., Álvarez R., Durán N.S., Illán R., González D.J. et al. Néstor-Guillermo progeria syndrome: A novel premature aging condition with early onset and chronic development caused by BANF1 mutations. Am. J. Med. Genet. Part A. 2011; 155:2617–2625. 10.1002/ajmg.a.34249 21932319

[40] Puente X.S. , Quesada V., Osorio F.G., Cabanillas R., Cadiñanos J., Fraile J.M., Ordóñez G.R., Puente D.A., Gutiérrez-Fernández A., Fanjul-Fernández M. et al. Exome sequencing and functional analysis identifies BANF1 mutation as the cause of a hereditary progeroid syndrome. Am. J. Hum. Genet. 2011; 88:650–656. 21549337 10.1016/j.ajhg.2011.04.010 PMC3146734

[41] Fisher H.G. , Patni N., Scheuerle A.E. An additional case of Néstor-Guillermo progeria syndrome diagnosed in early childhood. Am. J. Med. Genet. Part A. 2020; 182:2399–2402. 32783369 10.1002/ajmg.a.61777

[42] Eriksson M. , Brown W.T., Gordon L.B., Glynn M.W., Singer J., Scott L., Erdos M.R., Robbins C.M., Moses T.Y., Berglund P. et al. Recurrent de novo point mutations in lamin A cause Hutchinson-Gilford progeria syndrome. Nature. 2003; 423:293–298. 12714972 10.1038/nature01629 PMC10540076

[43] Vargas J.D. , Hatch E.M., Anderson D.J., Hetzer M.W. Transient nuclear envelope rupturing during interphase in human cancer cells. Nucleus. 2012; 3:88–100. 22567193 10.4161/nucl.18954 PMC3342953

[44] Chiang T.W.W. , Le Sage C., Larrieu D., Demir M., Jackson S.P. CRISPR-Cas9D10A nickase-based genotypic and phenotypic screening to enhance genome editing. Sci. Rep. 2016; 6:24356. 27079678 10.1038/srep24356 PMC4832145

[45] Janssen A.F.J. , Breusegem S.Y., Larrieu D. Current methods and pipelines for image-based quantitation of nuclear shape and nuclear envelope abnormalities. Cells. 2022; 11:347. 35159153 10.3390/cells11030347 PMC8834579

[46] Albanese S.K. , Parton D.L., Işık M., Rodríguez-Laureano L., Hanson S.M., Behr J.M., Gradia S., Jeans C., Levinson N.M., Seeliger M.A. et al. An open library of human kinase domain constructs for automated bacterial expression. Biochemistry. 2018; 57:4675–4689. 30004690 10.1021/acs.biochem.7b01081 PMC6081246

[47] Dimasi N. , Flot D., Dupeux F., Márquez J.A. Expression, crystallization and X-ray data collection from microcrystals of the extracellular domain of the human inhibitory receptor expressed on myeloid cells IREM-1. Acta Crystallogr. Sect. F Struct. Biol. Cryst. Commun. 2007; 63:204–208. 10.1107/S1744309107004903 PMC2330191 17329815

[48] Zander U. , Hoffmann G., Cornaciu I., Marquette J.-P., Papp G., Landret C., Seroul G., Sinoir J., Röwer M., Felisaz F. Automated harvesting and processing of protein crystals through laser photoablation. Acta Crystallogr Sect D Struct Biol. 2016; 72:454–466. 27050125 10.1107/S2059798316000954 PMC4822559

[49] Winn M.D. , Ballard C.C., Cowtan K.D., Dodson E.J., Emsley P., Evans P.R., Keegan R.M., Krissinel E.B., Leslie A.G.W., McCoy A. Overview of the CCP4 suite and current developments. Acta Crystallogr. Sect. D Biol. Crystallogr. 2011; 67:235–242. 21460441 10.1107/S0907444910045749 PMC3069738

[50] Vagin A. , Teplyakov A. MOLREP: an automated program for molecular replacement. J. Appl. Crystallogr. 1997; 30:1022–1025.

[51] Emsley P. , Lohkamp B., Scott W.G., Cowtan K. Features and development of Coot. Acta Crystallogr. Sect. D Biol. Crystallogr. 2010; 66:486–501. 20383002 10.1107/S0907444910007493 PMC2852313

[52] Bricogne G. , Blanc E., Brandl M., Flensburg C., Keller P., Paciorek W., Roversi P., Sharff A., Smart O.S., Vonrhein C. et al. BUSTER v2.10.3. 2020; Global Phasing Ltd.

[53] Adams P.D. , Afonine P. V, Bunkóczi G., Chen V.B., Davis I.W., Echols N., Headd J.J., Hung L.W., Kapral G.J., Grosse-Kunstleve R.W. et al. PHENIX: a comprehensive Python-based system for macromolecular structure solution. Acta. Crystallogr. D Biol. Crystallogr. 2010; 66:213–221. 20124702 10.1107/S0907444909052925 PMC2815670

[54] Vranken W.F. , Boucher W., Stevens T.J., Fogh R.H., Pajon A., Llinas M., Ulrich E.L., Markley J.L., Ionides J., Laue E.D. The CCPN data model for NMR spectroscopy: development of a software pipeline. Protein Struct. Funct. Bioinform. 2005; 59:687–696. 10.1002/prot.20449 15815974

[55] Loi M. , Cenni V., Duchi S., Squarzoni S., Otin C.L.-, Foisner R., Lattanzi G., Capanni C. Barrier-to-Autointegration Factor (BAF) involvement in prelamin A-related chromatin organization changes. Oncotarget. 2016; 7:15662–15677. 26701887 10.18632/oncotarget.6697 PMC4941268

[56] Freund A. , Laberge R.M., Demaria M., Campisi J. Lamin B1 loss is a senescence-associated biomarker. Mol. Biol. Cell. 2012; 23:2066–2075. 22496421 10.1091/mbc.E11-10-0884 PMC3364172

[57] Scaffidi P. , Misteli T. Reversal of the cellular phenotype in the premature aging disease Hutchinson-Gilford progeria syndrome. Nat. Med. 2005; 11:440–445. 15750600 10.1038/nm1204 PMC1351119

[58] Taimen P. , Pfleghaar K., Shimi T., Möller D., Ben-Harush K., Erdos M.R., Adam S.A., Herrmann H., Medalia O., Collins F.S. A progeria mutation reveals functions for lamin A in nuclear assembly, architecture, and chromosome organization. Proc. Natl Acad. Sci. 2009; 106:20788–20793. 19926845 10.1073/pnas.0911895106 PMC2779830

[59] Chen C.Y. , Chi Y.H., Mutalif R.A., Starost M.F., Myers T.G., Anderson S.A., Stewart C.L., Jeang K.T. Accumulation of the inner nuclear envelope protein Sun1 is pathogenic in progeric and dystrophic laminopathies. Cell. 2012; 149:565–577. 22541428 10.1016/j.cell.2012.01.059 PMC3340584

[60] Gonzalo S. , Kreienkamp R. DNA repair defects and genome instability in Hutchinson–Gilford progeria syndrome. Curr. Opin. Cell Biol. 2015; 34:75–83. 26079711 10.1016/j.ceb.2015.05.007 PMC4522337

[61] Shumaker D.K. , Dechat T., Kohlmaier A., Adam S.A., Bozovsky M.R., Erdos M.R., Eriksson M., Goldman A.E., Khuon S., Collins F.S. Mutant nuclear lamin A leads to progressive alterations of epigenetic control in premature aging. Proc. Natl Acad. Sci. 2006; 103:8703–8708. 16738054 10.1073/pnas.0602569103 PMC1472659

[62] Marcelot A. , Petitalot A., Ropars V., Le Du M.H., Samson C., Dubois S., Hoffmann G., Miron S., Cuniasse P., Marquez J.A. et al. Di-phosphorylated BAF shows altered structural dynamics and binding to DNA, but interacts with its nuclear envelope partners. Nucleic Acids Res. 2021; 49:3841–3855. 33744941 10.1093/nar/gkab184 PMC8053085

[63] Nichols R.J. , Wiebe M.S., Traktman P. The vaccinia-related kinases phosphorylate the N′ terminus of BAF, regulating its interaction with DNA and its retention in the nucleus. Mol. Biol. Cell. 2006; 17:2451–2464. 16495336 10.1091/mbc.E05-12-1179 PMC1446082

[64] Lin Q. , Yu B., Wang X., Zhu S., Zhao G., Jia M., Huang F., Xu N., Ren H., Jiang Q. et al. K6-linked SUMOylation of BAF regulates nuclear integrity and DNA replication in mammalian cells. Proc. Natl. Acad. Sci. 2020; 0:201912984. 10.1073/pnas.1912984117 PMC7229763 32332162

[65] Kono Y. , Adam S.A., Reddy K.L., Zheng Y., Medalia O., Goldman R.D., Kimura H., Shimi T. Nucleoplasmic lamin C rapidly accumulates at sites of nuclear envelope rupture with BAF and cGAS. 2022; bioRxiv doi:10 January 2022, preprint: not peer reviewed 10.1101/2022.01.05.475028. PMC9617480 36301259

[66] Krissinel E. , Henrick K. Protein interfaces, surfaces and assemblies service PISA at European Bioinformatics Institute. J. Mol. Biol. 2007; 372:774–797. 17681537 10.1016/j.jmb.2007.05.022

[67] Sears R.M. , Roux K.J. Mechanisms of A-type lamin targeting to nuclear ruptures are disrupted in LMNA- and BANF1-associated progerias. Cells. 2022; 11:865. 35269487 10.3390/cells11050865 PMC8909658

[68] Paquet N. , Box J.K., Ashton N.W., Suraweera A., Croft L. V., Urquhart A.J., Bolderson E., Zhang S.D., O’Byrne K.J., Richard D.J Néstor-Guillermo progeria syndrome: a biochemical insight into Barrier-to-Autointegration Factor 1, alanine 12 threonine mutation. BMC Mol. Biol. 2014; 15:27. 25495845 10.1186/s12867-014-0027-z PMC4266902

[69] Haraguchi T. , Kojidani T., Koujin T., Shimi T., Osakada H., Mori C., Yamamoto A., Hiraoka Y. Live cell imaging and electron microscopy reveal dynamic processes of BAF-directed nuclear envelope assembly. J. Cell Sci. 2008; 121:2540–2554. 18628300 10.1242/jcs.033597

[70] Fernandez A. , Bautista M., Wu L., Pinaud F. Emerin self-assembly and nucleoskeletal coupling regulate nuclear envelope mechanics against stress. J. Cell Sci. 2022; 135:jcs258969. 35178558 10.1242/jcs.258969 PMC8995096

